# Segmentation, tracking and cell cycle analysis of live-cell imaging data with Cell-ACDC

**DOI:** 10.1186/s12915-022-01372-6

**Published:** 2022-08-05

**Authors:** Francesco Padovani, Benedikt Mairhörmann, Pascal Falter-Braun, Jette Lengefeld, Kurt M. Schmoller

**Affiliations:** 1 Institute of Functional Epigenetics (IFE), Molecular Targets and Therapeutics Center (MTTC), Helmholtz Center Munich, 85764 Munich-Neuherberg, Germany; 2 Institute of Network Biology (INET), Molecular Targets and Therapeutics Center (MTTC), Helmholtz Center Munich, 85764 Munich-Neuherberg, Germany; 3 Microbe-Host Interactions, Faculty of Biology, Ludwig-Maximilians-University (LMU) München, 82152, Planegg-, Martinsried, Germany; 4 Institute of Biotechnology, HiLIFE, University of Helsinki, Biocenter 2, P.O.Box 56 (Viikinkaari 5 D), 00014 Helsinki, Finland; 5 Department of Biosciences and Nutrition (BioNut), Karolinska Institutet, Huddinge, Sweden; 6 German Center for Diabetes Research (DZD), 85764, Munich-Neuherberg, Germany

**Keywords:** Live-cell imaging, Deep-learning cell segmentation, Cell tracking, Cell cycle analysis, Bioimage analysis

## Abstract

**Background:**

High-throughput live-cell imaging is a powerful tool to study dynamic cellular processes in single cells but creates a bottleneck at the stage of data analysis, due to the large amount of data generated and limitations of analytical pipelines. Recent progress on deep learning dramatically improved cell segmentation and tracking. Nevertheless, manual data validation and correction is typically still required and tools spanning the complete range of image analysis are still needed.

**Results:**

We present Cell-ACDC, an open-source user-friendly GUI-based framework written in Python, for segmentation, tracking and cell cycle annotations. We included state-of-the-art deep learning models for single-cell segmentation of mammalian and yeast cells alongside cell tracking methods and an intuitive, semi-automated workflow for cell cycle annotation of single cells. Using Cell-ACDC, we found that mTOR activity in hematopoietic stem cells is largely independent of cell volume. By contrast, smaller cells exhibit higher p38 activity, consistent with a role of p38 in regulation of cell size. Additionally, we show that, in *S. cerevisiae*, histone Htb1 concentrations decrease with replicative age.

**Conclusions:**

Cell-ACDC provides a framework for the application of state-of-the-art deep learning models to the analysis of live cell imaging data without programming knowledge. Furthermore, it allows for visualization and correction of segmentation and tracking errors as well as annotation of cell cycle stages. We embedded several smart algorithms that make the correction and annotation process fast and intuitive. Finally, the open-source and modularized nature of Cell-ACDC will enable simple and fast integration of new deep learning-based and traditional methods for cell segmentation, tracking, and downstream image analysis.

Source code: https://github.com/SchmollerLab/Cell_ACDC

**Supplementary Information:**

The online version contains supplementary material available at 10.1186/s12915-022-01372-6.

## Background

Live-cell imaging is a powerful technique that allows studying complex cellular dynamics by providing spatiotemporal information of subcellular events [1]. Microfluidic devices that maintain constant environments enable parallel imaging of thousands of cells for many hours in a single experiment. However, downstream analysis typically involves many potentially time-consuming steps, e.g. cell segmentation, tracking, and pedigree annotation. Thus, for the large amount of data typically produced by a live-cell imaging experiment, downstream extraction of biologically relevant information becomes the rate-limiting step.

While traditional segmentation algorithms had low generalization power, recent advances in deep learning, and specifically in fully convolutional neural networks (FCNN) based on U-Net [2], have greatly enhanced segmentation accuracy and degree of automation [3, 4]. More specifically, in the case of live-cell microscopy of yeast and other organisms (e.g. mammalian stem cells), neural networks recently published (YeaZ [5], Cellpose [6], YeastMate [7] and StarDist [8]) drastically improved the segmentation process. However, even these neural networks do not achieve perfect segmentation, and—depending on the question—manual verification or correction of segmentation and tracking is often still essential for high-quality microscopy image analysis. Additionally, training deep learning models requires annotated ground-truth data.

Single-live-cell timelapse microscopy enables the study of cellular events happening in different phases of the cell cycle or even across multiple cell cycles. For this purpose, analysis of movies that image cells over multiple generations requires the correct annotation of pedigrees and cell cycle transitions. This is particularly true for budding yeast, because the bud, even though still a part of the mother cell, needs to be segmented as an individual object. This is important because bud emergence marks S-phase entry, a key cell cycle transition. In addition, the bud needs to be separated from the mother to answer scientific questions related to the transport of sub-cellular components between the mother and the bud. Furthermore, volume estimation (see “Cell volume calculation” in the Material and methods section), requires separate mother and bud segmentation masks. To obtain information about a full cell cycle, it is then necessary to link a bud to its mother cell and determine the time point of cell division. Importantly, budding yeast cell cycle annotations can in part be performed in a label-free manner based on the phase contrast signal: bud emergence is linked to S-phase entry, and cell division is typically detectable by a sudden movement of the bud that is not mechanically linked to the mother cell anymore. Unfortunately, such pedigree and cell cycle annotations in budding yeast involve many manual steps (without a dedicated fluorescent marker) that require careful inspection of every single frame to identify and annotate the time point of cell division. Fluorescent tagging of proteins that locate to the bud neck connecting mother and bud, or of histones to monitor S-phase and observe nuclear localization, facilitates pedigree annotation and has been used for automation [9–12]. However, endogenous tagging comes with the cost of requiring genetic manipulation as well as one fluorescent channel that otherwise could be used for other purposes. Automated approaches do not achieve the close-to-perfect accuracy required for many questions and thus still require manual inspection and correction. While tools have been previously developed for automatic lineage tree construction [13–18] they are specific for symmetrically dividing cells or require a dedicated fluorescent marker for the cell cycle stage inference.

Although many software tools dedicated to the analysis of live-cell microscopy have been developed in the past (ImageJ/Fiji [19], MorphoLibJ [20], PhyloCell [21], CellProfiler [22], Cell Tracer [23], Wood et al. [24, 25], Cell Star [26], Cell Serpent [27], Tracker [28], YeastSpotter [29], YeastNet [30], DeepCell [31], Cellbow [32], LABKIT [33], largely focussed on classification tasks CellID [34] and Advanced Cell Classifier [35] and, specifically for ageing experiments using dedicated microfluidics, DISCO [36], DetecDiv [37] and BABY [38]), to the best of our knowledge, none of them spanned the entire image analysis pipeline from CNN-based segmentation to cell cycle analysis in growing colonies, and fluorescent signal quantification (Table 1).

**Table 1:**
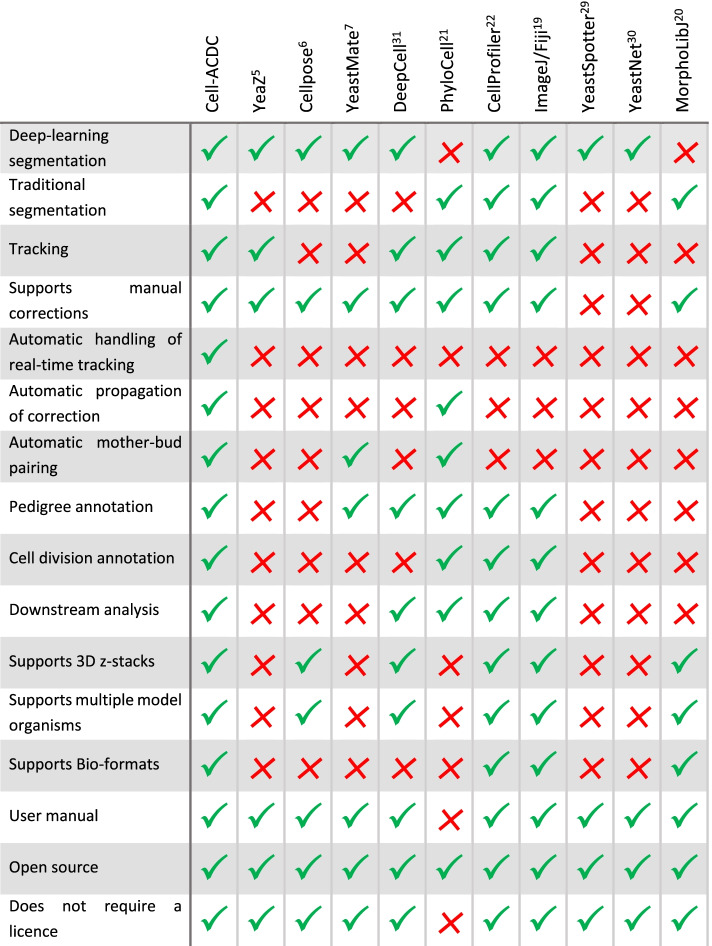
Comparison between Cell-ACDC and other available software

*Automatic handling of real-time tracking*: Cell-ACDC has real-time tracking to aid with the correction process and it automatically detects which frame was already visited and corrected to avoid that wrong tracking invalidates that frame again. Note that the table contains only software that either uses a deep-learning approach or includes tracking and downstream analysis of growing cell populations.

### Implementation

Here we present an open-source graphical user interface (GUI)-based framework (written in Python 3) embedding state-of-the-art neural networks (YeaZ [5], Cellpose [6], StarDist [8] and YeastMate [7]) selectable by the user and smart algorithms that allow for fast, replicable, and accurate microscopy image analysis. The provided tools cover the entire image analysis pipeline from a raw microscopy file to the quantification of the feature of interest. We named this software Cell-ACDC for Cell-Analysis of the Cell Division Cycle.

Cell-ACDC was developed following a community-centred approach, where users from several research groups provided feedback and suggestions that were implemented into the pipeline. Additional segmentation models that will be developed in the future can be easily added in a few minutes with a drop-in approach. Cell-ACDC provides for the first time the possibility to constantly visualize and correct any segmentation, tracking, or cell cycle annotation error in a fast and intuitive way. It includes several smart algorithms and shortcuts that automatically propagate any change to past and future frames to continuously maintain data integrity and correctness. In essence, Cell-ACDC is a modular framework for cell segmentation, tracking, and cell cycle analysis that enables researchers to achieve near 100% accuracy in a reasonable amount of time. We designed Cell-ACDC by complementing the best tools existing with a complete image analysis workflow, a process that can otherwise take months to develop for each specific research question. Finally, by standardizing handling and analysis of live-cell microscopy data, Cell-ACDC facilitates data sharing between different labs.

One key advantage of Cell-ACDC is that complete pedigrees over multiple cell divisions can be obtained with reasonable manual effort. This allowed us to quantify histone Htb1 protein concentrations in budding yeast over multiple cell cycles, revealing that Htb1 concentrations decrease with replicative cell age. Moreover, going beyond the analysis of budding yeast, we used Cell-ACDC to study regulatory pathways controlling cell size and growth in hematopoietic stem cells. We found that while mTOR activity is largely constant as a function of cell size, p38 activity is higher in small cells, consistent with a role of p38 in controlling cell-size-dependent cell cycle progression [39].

**Fig. 1:**
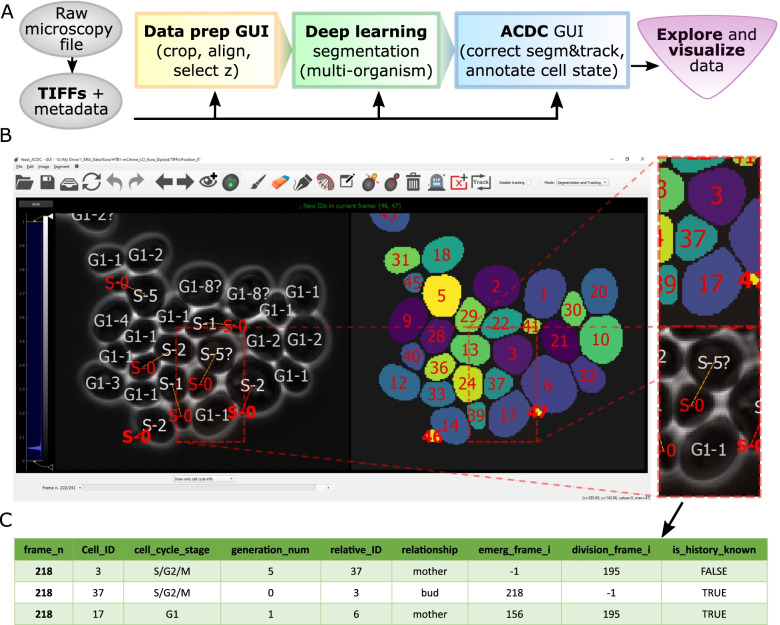
Overview of pipeline and GUI. **A** Flowchart representation of the Cell-ACDC pipeline. In the first step, the raw microscopy file(s) is/are automatically converted into TIFFs, the relevant metadata is extracted, and the files are arranged in the data structure required by Cell-ACDC. Next, the user can launch any of the three main modules: (1) GUI-based data prep where the user can align time-lapse data, select a z-slice or a projection for 3D z-stacks data, and/or crop data to reduce memory usage; (2) automatic segmentation/tracking of multiple positions and/or multiple time-frames (batch-processing) using the embedded neural network models. (3) **B** Main user interface, where the user visualizes and corrects the result of automatic segmentation and tracking. Almost all the available functions (such as brush, eraser, edit ID or auto-separate cells) are easily accessible from a button on the top toolbar, while sliders under the left image allow quick visualization of a specific position, frame, or z-slice. To enhance visualization of the signal in the left image, the user can adjust the intensity levels with two vertical sliders on the left side of the GUI. **C** Example of the output table generated by cell cycle annotations. The annotations are saved in CSV format allowing for quick import into GUI- or script-based spreadsheets software. The information saved includes the frame number, the cell ID, the cell cycle stage (either “G1” or “S/G2/M”), the generation number (automatically increased when division is annotated), the relative ID of the assigned parent cell, the relationship with the relative ID (either “mother” for both mother cells and cells in G1, or “bud” for buds that did not divide yet), the frame when the cell emerged and divided, and whether the history of the cell is fully known or not. Examples of cells with history not fully known are cells already present at frame 1 and cells appearing at a specific time point from outside of the field of view. Note that “is_history_known” is also visually highlighted with a question mark on the cell (e.g. cell ID 3, which was present at frame 1)

Cell-ACDC provides a framework that allows the implementation of the entire image analysis pipeline, from raw microscopy files to visualizing results (Fig. 1A). In the first steps, the raw microscopy files are converted into TIFF files (one for each channel of each position) using the popular Bio-Formats [40] library in a fully automated Python routine (opening, reading, and converting from raw microscopy files is performed by a dedicated Cell-ACDC sub-module). Using Bio-Formats allows for standardized reading of the file metadata, such as the number of frames, the number of z-slices in a z-stack, or the time interval between each frame etc. Furthermore, we provide full support for 2D, 3D (single z-stacks or 2D images over time) and 4D images (3D z-stacks over time) with multiple channels and multiple positions. Note that the TIFF format was chosen for its widespread use and compatibility with popular image viewers such as Fiji and napari [29].

After the conversion of the image file format, the user can select any of the three following steps: (a) a GUI for multiple data preparation steps (aligning frames, cropping images, determining the area for background noise calculation, and selecting z-slice or projection for the segmentation step), (b) automated segmentation and tracking using state-of-the-art neural networks and (c) a GUI for semi-automated correction of segmentation and tracking errors supporting diverse model organisms/objects, plus annotations of budding yeast cell cycle and pedigrees (Fig. 1B and Additional file 1 - Movie).

While it is possible to perform segmentation for single frames in the GUI, we highly recommend using the dedicated segmentation and tracking script for whole batches. We embedded four neural networks that were recently published: YeaZ [5] and YeastMate [7] for yeast cells, and Cellpose [6] and StarDist [8] for multiple model organisms (bright-field and phase contrast). The modularity of the code allows for easy and quick implementation of any other segmentation algorithm (traditional or deep-learning-based).

Alongside segmentation and tracking functionalities, the GUI has an additional working mode: pedigree and cell cycle annotations. These functionalities were specifically developed for the cell cycle analysis of budding yeast cells but can be adapted to other model organisms in the future to handle symmetric cell division as well. Annotations of the yeast cell cycle include two main steps: (a) assigning the bud to the correct mother cell and (b) annotating the cell division event. Annotations are stored in a tabular format (Fig. 1C) that allows reconstruction of the entire pedigree of each single cell and downstream extraction of data of interest.

Independent of whether the user decides to use the cell cycle annotation functionality, Cell-ACDC produces comprehensive output data on a single-cell level. The extraction of metadata from raw files mentioned above allows for the approximation of volumes based on the segmentation masks. Analysis of additional (fluorescence) channels enables the calculation of several quantities, such as amount, concentration, or median signal strength of the fluorescent markers. For this step, we provide an Application Programming Interface (API) including calculations of custom metrics. Finally, the annotation of the cell cycle additionally allows analysis of those quantities in the context of the cell cycle and calculation of time-dependent properties such as growth rates.

## Results

### Overview of functionalities

**Fig. 2:**
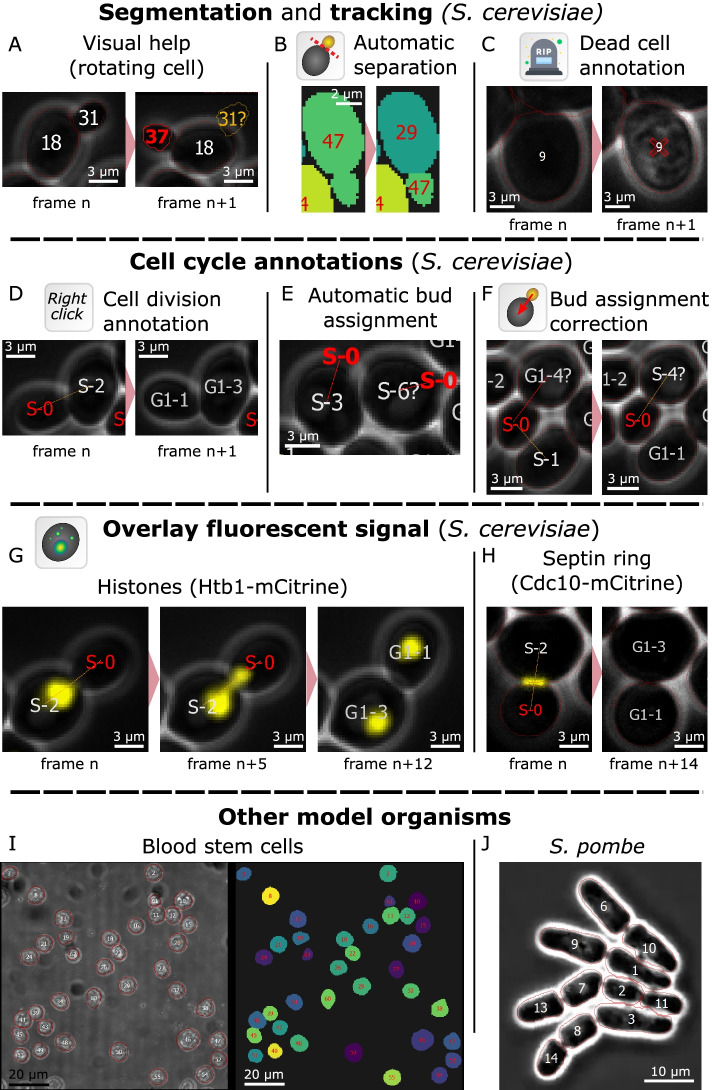
Examples of Cell-ACDC functions. **A** Visual help: information such as the cell ID, the cell cycle stage, and the generation number, as well as the segmentation contour are conveniently displayed on the cell image. Information is colour-coded: red for newly emerged/appeared cells, white for cells already present in the previous frame, and yellow for disappeared cells. This allows for quick identification of tracking errors since often lost cells are caused by an ID misplaced due to the tracking algorithm failing. **B** Automatic separation: With a single click on the merged cells, the user can trigger automatic separation. With a combination of convexity defects detection and contour approximation, the algorithm separates the cells along the predicted plane. **C** Annotate cell as “dead”: A cell can be annotated as dead with a single click, and it is then considered dead for all future frames. The user can always annotate the cell as not dead at any point in future frames. **D** Annotate cell division: Cell division is often visible due to a sudden movement of the bud. The user can then click on the cell that divided to annotate it. The related information, such as generation number and cell cycle stage, is then automatically updated for both the mother and daughter cell. This annotation can be undone at any time point in past or future frames and all the information in all the involved frames is automatically updated. **E** Automatic mother-bud pairing: When a new cell appears, an automatic assignment algorithm is triggered. Using a cost-optimization routine, the new cell is assigned to the predicted mother. **F** Mother-bud pairing correction: When the automatic mother-bud pairing fails, the user can correct the assignment with a drag and drop gesture. This can be done at any time-point of the life of the new cell and the pairing is automatically corrected on all the relevant past and future frames. **G** Overlay fluorescent signal from tagged histone Htb1. If available, the user can overlay a fluorescent signal. This is helpful, if, for example, the tagged gene is a cell cycle marker that can aid cell cycle annotations. **H** Overlay fluorescent signal from tagged septin ring (Cdc10). **I** Representative images of murine hematopoietic stem cells segmented based on bright-field signal using Cell-ACDC (based on Cellpose, using the median z-projection). **J** Segmentation using Cell-ACDC (based on YeaZ) of fission yeast (*S. pombe*). Data from [41]

The recent advancement in deep-learning-based segmentation algorithms greatly reduced the segmentation error rate, but unfortunately many times it is still required to visually inspect and correct these errors. This is a tedious and time-consuming process, especially for live-cell imaging experiments where an error in one frame requires correction of all the future frames (often hundreds of frames, see “Benchmarking” in the “Materials and methods” section). For this specific step, the GUI needed to be fast, intuitive, responsive, and interactive. To allow easy detection of potential errors, we included visual help directly displayed on the images and segmentation masks while navigating through the frames (Fig. 2A and Additional file 2 - Movie) including cell contours, cell ID, cell cycle information, as well as lost and newly appearing cells’ contours. We automated the propagation of manual corrections to future and past frames along with continuous tracking of the segmented cells while maintaining consistency with already annotated parts of the data. We implemented automated and semi-automated functions to allow quick and accurate correction once an error is detected. To simplify the correction of segmentation errors we embedded traditional segmentation algorithms, such as random walker and flood fill, alongside manual tools such as brush and eraser. Using the segmentation masks, Cell-ACDC also computes several single-cell numerical features based on the segmentation of any loaded fluorescent channel. These features include cell area, estimated cell volumes (see “Cell volume calculation” in the “Materials and methods” section), alongside mean, maximum, median and quantiles of the fluorescent signal. To visualize and interactively explore the data produced, we provide Jupyter notebooks (see “Downstream analysis” in the “Materials and methods” section). While we only highlight a few examples here, we explain each function in detail in the manual (Supplementary information).

A typical time-consuming correction is editing the ID of an object when tracking fails. Since most of the tracking algorithms track objects based only on the previous frame, a tracking error at one frame results in the error being propagated through all preceding frames in the video. The Cell-ACDC GUI provides a real-time tracking mode that is activated when browsing through unseen frames (see “Continuous tracking” in the “Materials and methods” section). This allows for seamless correction of tracking errors while analysing the video frame-by-frame. Another typical segmentation error occurs when two cells are segmented as a single object (usually a mother cell with a small bud, Fig. 2B and Additional file 3 - Movie). For this specific case, we developed a custom algorithm for the automatic separation of the merged cells. Based on a combination of convexity defects detection and contour approximation, the cells are automatically separated. We compared this method to classic distance transform followed by watershed separation that is implemented in YeaZ, and we found consistently better performance in these specific cases (Additional file 4: Fig. S1). Note that this function is triggered by the user with a mouse click on the cells that requires separation. If automated separation fails, the user can separate the cells manually with a dedicated function. An additional fundamental requirement is the possibility to annotate images. We implemented a variety of functionalities to annotate specific cell states, such as “dead” (Fig. 2C) or “excluded from the analysis” that are activated with a single click on the cell. The corrected annotation is automatically propagated to all future frames and can be undone at any time point.

After acquiring time-lapse microscopy data of proliferating budding yeast, a typical analysis involves annotating budding events, division events, and identifying mother-bud pairs (Additional file 6 - Movie). For these specific steps, we developed three main actions: (a) annotation of cell division time point (Fig. 2D), (b) automatic bud assignment (Fig. 2E, and “Automatic mother-pairing” in the “Materials and methods” section), and (c) semi-automated bud assignment correction (Fig. 2F, and “Automatic propagation of corrections to future and past frames” in the “Materials and methods” section).

Without the use of a cell cycle marker, cell division is often visible in phase-contrast images due to a sudden movement of the bud. To annotate this event, the user simply clicks once on the mother or bud. Cell-ACDC will automatically update the annotations table by changing the cell cycle stage and increasing the generation number to keep track of how many times a cell budded. Many times, this event is clearly visible, but other times it requires careful inspection to spot a subtle movement indicating cell division. To spot the event in this case, the user must constantly jump back and forth between frames. Therefore, responsiveness and speed of displaying data are fundamental. To achieve this, we used the high-performance python library *PyQtGraph* for the GUI elements*.* Furthermore, in practice, it is often necessary to correct a cell division annotation multiple times. Therefore, Cell-ACDC automatically propagates corrections to all involved frames.

An important objective of cell cycle analysis with budding yeast is the assignment of newly emerging buds to the correct mother cell. Using a cost optimization routine, Cell-ACDC automatically assigns each emerging bud to the predicted mother cell. For all new buds, the algorithm calculates the cost of assigning the bud to any cell in G1 (i.e., cells that are not budding now). For two cells, the cost is defined to be the single linkage distance between the cells’ pixels. This cost is then minimized using a modified Jonker-Volgenant algorithm with no initialization [42]. The function solves a minimum cost matching problem where we define all new buds as the one and all G1 cells as the other bipartite set which are matched to each other. To quantitatively benchmark mother-bud pairing accuracy (percentage of correctly assigned buds), we tested the algorithm with time-lapse data in three different scenarios: (a) data automatically segmented with YeaZ without any correction of segmentation and tracking errors, where all the cells are eligible mothers; (b) data with correction of segmentation and tracking errors, where all the cells are eligible mothers; and (c) data with correction, where only cells in G1 are eligible mother cells. Note that (c) is the scenario in which the algorithm is currently used. In scenario (a) and (b), we obtained an accuracy of 67.5% and 75.5% (*n*=120) respectively. In scenario (c), we obtained an accuracy of 90.5% (*n*=147). Note that to achieve 90.5% accuracy, prior knowledge of which cells are in G1 in the previous frame is required, and the mother-bud assignment is automatic between two consecutive frames and not the entire video. In some cases, the assignment fails, e.g. when a bud emerges close to another cell in G1 that is not the mother cell, or due to errors in earlier frames (e.g. when the correct mother cell is not annotated as being in G1). Three common scenarios that could result in wrong annotations on the next time-point are (1) buds that, as soon as they separate from the mother, are washed away from the field of view without giving the user the possibility to correctly annotate the first frame after cell division; (2) not enough cells in G1 for the number of new cells appearing (potential buds); and (3) trying to assign a bud to a cell in G1 that already has a bud assigned to it in the relevant past and/or future frames (it would result in a mother cell with two buds assigned to it). All these scenarios are automatically detected by Cell-ACDC and the user is notified with a dialogue that allows choosing the best course of action. Moreover, it is sometimes not possible to determine the correctness of the assignment on the current frame, and the correct pairing is visible only after the bud has increased its size. Manually correcting such assignments would require correcting many frames where the bud must be assigned to another cell in G1 and reverting the wrong mother’s cell cycle stage back to G1. Again, automated correction propagation is a key feature that facilitates rapid annotation.

Additionally, while it is possible to annotate the cell cycle stage using only phase contrast signal, this step can be facilitated by a fluorescent marker, such as tagged histone (e.g. Htb1 in yeast, Fig. 2G) to follow the segregation of the nucleus from the mother to the bud [43], or the septin ring (e.g. Cdc10, Fig. 2H) to determine cytokinesis events [44]. To allow visualization of such fluorescent cell cycle markers, we implemented an overlay function, activated using a button on the toolbar.

Finally, Cell-ACDC also serves as a framework for the segmentation/tracking of other organisms such as hematopoietic stem cells (Fig. 2I) or the fission yeast *S. pombe* (Fig. 2J). Testing Cell-ACDC on fission yeast, we found that also for symmetrically dividing cells, cell cycle annotations and pedigree analysis are possible: after division, the two daughter cells are automatically paired, and through tracking linked to their predicted mother cell. The user can use the already implemented features to annotate division and mother-daughter pairing.

### Validation of the image analysis pipeline

**Fig. 3:**
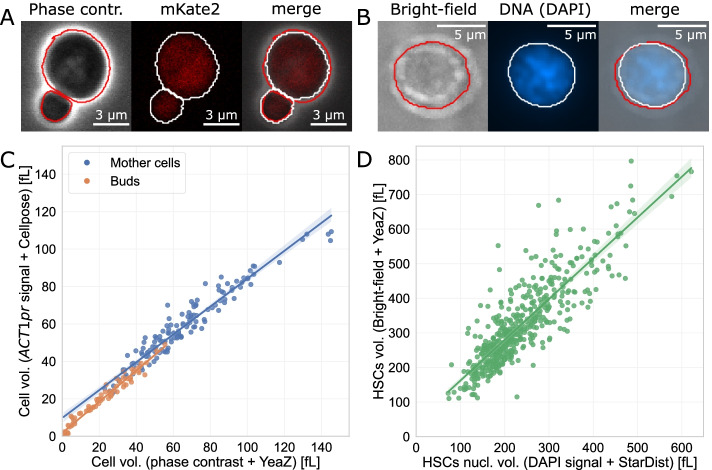
Cell-ACDC analysis pipeline validation. **A** Representative images of the strain carrying *ACT1pr-mKate2*. **B** Representative image of hematopoietic stem cells from wild-type mice stained for DNA (DAPI). **C** Correlation between budding yeast cell volume calculated from cells segmented with two different methods. The cell volume is calculated from 2D segmentation masks (see [45] and the “Materials and methods” section), which were obtained with two different methods: segmentation with YeaZ on phase-contrast signal and segmentation with Cellpose on the fluorescent signal of mKate2 expressed from an additional *ACT1* promoter. We observe a strong agreement between the two methods (*n* = 113, Pearson’s coefficient=0.98, *p* value<10^−10^). **D** Correlation between the nucleus and cell volume of hematopoietic stems cells (HSCs). For the nuclear volume, we segmented the DAPI signal using StarDist, while for the cell volume, we segmented the bright-field channel using YeaZ. We observe high correlation (*n* = 519, Pearson’s coefficient=0.86, *p* value <10^−10^), indicating that the nuclear volume is a valid proxy for cell size. All segmentation masks were carefully inspected and corrected using Cell-ACDC

Cell-ACDC offers full support for the segmentation and analysis of 3D z-stacks. Furthermore, together with the neural network Cellpose and StarDist, it is possible to segment cells of various model organisms other than budding yeast. To validate the entire image analysis pipeline including the use of 3D z-stacks, we first analysed single time-point images of budding yeast. Using a strain expressing the fluorescent protein mKate2 from an *ACT1* promoter, we imaged both phase contrast and mKate2 signal (Fig. 3A). Secondly, using the Data Prep GUI (automatically called when segmenting 3D z-stacks), we visually selected the optimal z-slices or the projection mode. Thirdly, we segmented cells (using batch processing capabilities of Cell-ACDC) in the phase-contrast signal using the neural network YeaZ, and the mKate2 signal using Cellpose. Finally, we calculated the cell volume (see the Materials and methods” section) for both segmentations. We found a strong correlation between the cell volumes calculated with the two methods (Fig. 3C, Pearson’s correlation = 0.98, *p* value <10^−10^), indicating a strong match between cell volume estimates obtained from two different channels using two different neural networks.

To validate the capabilities of Cell-ACDC to segment other model organisms, we segmented both the nucleus (DAPI staining) and the bright-field channel of hematopoietic stem cells (HSCs). Benefitting from the flexibility of using multiple deep learning segmentation models, we chose StarDist to segment the DAPI channel and YeaZ for the bright-field channel. We then carefully inspected and corrected segmentation errors and computed cell and nuclear volumes using the Cell-ACDC main GUI. Consistent with previous reports [46–48], we found that the nucleus occupies a large fraction of the cell volume, and that nucleus and cell volume are well correlated (Fig. 3B, D, Pearson’s correlation = 0.86, *p* value<10^−10^). Our results demonstrate that for HSCs, both nucleus and cell volume are valid proxies for cell size.

### A role of p38 MAPK pathways in cell size regulation of hematopoietic stem cells

**Fig. 4:**
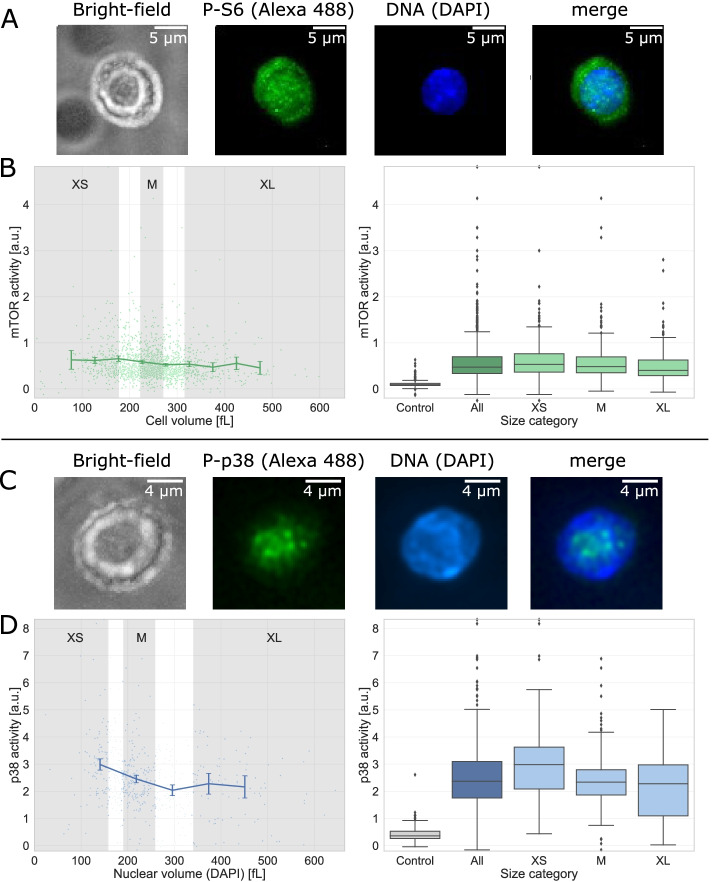
mTOR and p38 activity in relation to cell size. **A** Representative image of hematopoietic stem cells from wild-type mice stained for Phospho-S6 (P-S6, Alexa 488) and DNA (DAPI). **B** Values obtained from Cell-ACDC segmentation using Cellpose on bright-field signal: Alexa 488 total fluorescence intensity per cell volume (a.u., proxy for mTOR activity) of phospho-S6 ribosomal protein (Ser240/244) staining as a function of the HSC volume (fL). Gates of XS-, M- and XL-sized HSCs are indicated in grey (*n*=1626 cells). Alexa 488 total fluorescence intensity per cell volume (a.u.) of phospho-S6 ribosomal protein (Ser240/244) staining in control (no primary antibody), all HSCs, XS-sized HSCs (*n*=247), M-sized HSCs (*n*=493), and XL-sized HSCs (*n*=244). **C** Representative image of hematopoietic stem cells from wild-type mice stained for Phospho-p38 (P-p38, Alexa 488) and DNA (DAPI). **D** Values obtained from Cell-ACDC segmentation (using StarDist on DAPI signal): Alexa 488 total fluorescence intensity per nuclear volume (a.u., proxy for p38 activity) of Phospho-p38 mitogen-activate protein kinase (MAPK, Thr180/Tyr182) staining as a function of the HSC nuclear volume (fL). Gates of XS-, M- and XL-sized HSCs are indicated in grey (*n* = 586). Alexa 488 total fluorescence intensity per cell volume (a.u.) of phospho-p38 (Thr180/Tyr182) staining in control (no primary antibody, *n* = 196), all HSCs, XS-sized HSCs (*n* = 90), M-sized HSCs (*n* = 179), and XL-sized HSCs (*n* = 77)

Cells need to accurately control their size to maintain cellular functions [46, 49]. In particular, increased cell size can impair the function of stem cells [50]. Nevertheless, little is known about how stem cells regulate their size. To carefully quantify factors involved in size regulation in hematopoietic stem cells (HSCs), we validated the capabilities of Cell-ACDC to segment stem cells and extract automatically calculated metrics from the fluorescent signal to provide novel biological insights. First, we segmented hematopoietic stem cells (HSCs) from an immunofluorescence staining for phospho-S6 ribosomal protein (Ser240/244) using bright-field images to determine cell volume (Fig. 4A). Based on this segmentation, we evaluated the total Alexa 488 fluorescence intensity divided by cell volume, which is a readout for mTOR activity (Fig. 4B). Our results validate manual measurements showing that mTOR activity stays largely constant with increasing HSC volume [46]. This result supports a previously proposed model that changes in cell cycle length, rather than variations in mTOR activity, affect HSC size [46]. Next, we focused on another factor, p38 mitogen-activated protein kinase (MAPK), which was previously associated with cell size regulation by (i) affecting G1 duration [39] and (ii) regulating ion channel permeability [51]. As a readout for p38 activity in HSCs, we used immunofluorescence staining of phosphor-p38 (Thr180/Tyr182). We found the signal to be nuclear, and therefore decided to normalize the total fluorescence intensity on the nuclear volume we obtained by segmenting the nucleus (DAPI staining, Fig. 4C). We found that small HSCs display higher p38 activity (Fig. 4D), which is in line with previous findings suggesting that increased p38 activity in small RPE1 cells prolongs their G1 phase, allowing cells to grow to their optimal size [39, 52]. Overall, these results demonstrate that Cell-ACDC enables the reliable and efficient analysis of fluorescence images of murine stem cells. Furthermore, our data support models suggesting that control of cell cycle duration is a major mechanism of stem cells to regulate their size.

### Image analysis of single-live-cell imaging experiments

**Fig. 5:**
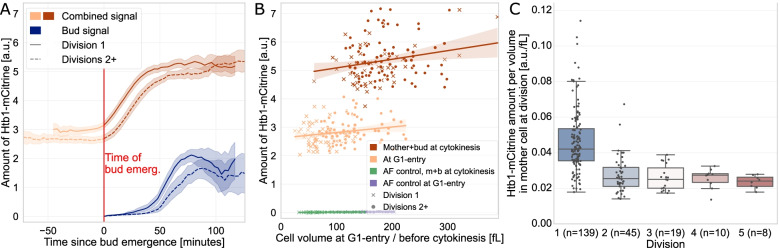
Quantitative analysis of Htb1-mCitrine expression as a function of cell cycle and cell volume. **A** Total amount of Htb1-mCitrine (total cellular fluorescence intensity after background subtraction) as a function of time for the first cell cycle of new-born daughter cells (*n*=48) and older cells (*n*=89). Single cell traces are aligned at bud emergence (time = 0). **B** Amount of Htb1-mCitrine as a function of cell volume at birth (blue) and directly before cytokinesis (combined signal of mother and bud, orange). Signal from untagged strains used as autofluorescence (Af) control is negligible. Results from **A** and **B** are consistent with our previous analysis [35]. **C** Concentration of Htb1-mCitrine retained in mother cells at cell division, shown as a function of division number (boxplot whiskers: 1.5 IQR)

To validate the image analysis pipeline for live-cell imaging assays, we re-analysed time-course images of a yeast strain expressing the histone Htb1 endogenously tagged with a fluorescent reporter (mCitrine) that we have previously analysed using a dedicated custom Matlab-script [25, 53]. Histones are expressed in a cell cycle-dependent manner, with expression tightly coupled to DNA synthesis during S-phase [54]. After aligning the frames with the Data Prep GUI to correct for shifts during the time-lapse experiment, we segmented the videos with the batch processing segmentation script, using YeaZ on the phase-contrast signal. Next, we corrected segmentation and tracking errors, and we annotated cell cycle progression in the main GUI. Finally, we implemented a notebook in the popular open-source web application Jupyter Notebook [55] to allow interactive transformation, exploration, statistical analysis and visualization of the Cell-ACDC output data. As expected [53], by plotting the Htb1-mCitrine amount over entire cell cycles aligned at bud emergence, we observe a strong cell cycle dependence of Htb1 expression and a 2-fold increase around DNA replication (Fig. 5A, *n*=137). Cell cycle annotations also allow comparing results at different cell cycle stages. We show that the amount of Htb1-mCitrine in single mother-bud pairs (before division) is about double the amount in single cells at birth (start of G1 phase). Moreover, confirming our previous analysis, we find that the amount of histones at a given cell cycle stage is largely independent of cell volume [53] (Fig. 5B). By additional analysis of an untagged control strain, we ensured that autofluorescence is negligible compared to the histone-specific fluorescence signal. A key advantage of Cell-ACDC compared to our previous analysis is that it now allows us to obtain full pedigrees spanning the complete duration of the experiment. Thus, going beyond our previous analysis, Cell-ACDC enabled us to separately analyse new-born daughter cells during their first cell cycle as well as older mother cells. We found that at a given cell volume, histone amounts in new-born cells during their first cell cycle are similar to those in older cells during later cell cycles (Fig. 5A, B). We then quantified the concentration of Htb1-mCitrine in mother cells at the beginning of G1 as a function of replicative age. We found that Htb1-mCitrine concentrations decrease with increasing division number (Fig. 5C), in particular after the first complete cell cycle which results in a pronounced increase of cell volume. Taken together, our results demonstrate that at least during the first cell cycles, cells maintain roughly constant amounts of histones, leading to a decrease of histone concentration due to cell growth. This suggests that the increase of cell size could in part account for the reported decrease of histone concentrations during replicative ageing [45].

## Discussion

Analysis of live-cell imaging data is a complex task that involves several steps, some of which are often laborious and time-consuming. Despite great advances in image analysis algorithms, such as convolutional neural networks, extracting useful biological information from microscopy images can require the implementation of sophisticated pipelines. Here, we presented Cell-ACDC, an open-source, GUI-based framework that enables fast, accurate and intuitive analysis of microscopy images. We provide tools for each step of the pipeline, from the raw microscopy file to the visualization of the results (Fig. 1). The software is written in Python, which is freely available for all users. We embedded recent neural network models for object detection and image segmentation, YeaZ, Cellpose, StarDist and YeastMate. While YeaZ and YeastMate were specifically developed for the segmentation of yeast cells, Cellpose and StarDist are generalist, enabling the segmentation of multiple model organisms.

Cell-ACDC can analyse images with different dimensionalities, from a single 2D image to 3D (z-stacks or 2D images over time) and 4D images (3D z-stacks over time). We implemented building blocks which can be arranged to workflows tuned to each specific image type.

For time-stacks, we provide a set of tools for single-cell tracking and annotation of the yeast cell cycle. Despite the great accuracy of the embedded segmentation models, it is often required to visually inspect and correct segmentation and tracking errors. Cell-ACDC was developed to enable fast and intuitive correction of these errors, with automatic handling of correction propagation to past and future frames (Fig. 2). For budding yeast live-cell imaging assays, we implemented a workflow to enable annotation of the cell cycle, either from phase-contrast signal or from a fluorescent cell cycle marker (e.g. Htb1, Cdc10). With a combination of automatic mother-bud pairing and semi-automatic cell division annotation, Cell-ACDC enables accurate and fast annotation of the cell cycle stage for pedigree analysis.

For z-stacks, the user can select a specific z-slice or a projection to use for segmentation. Converting a z-stack into a 2D image is required for segmentation in Cell-ACDC. Note that Cellpose supports segmentation of 3D z-stacks directly, however, images of yeast cells (bright-field and phase contrast) usually contain only a few z-slices that are in-focus and therefore usable for segmentation. Additionally, 3D segmentation with neural networks is often computationally more expensive than the 2D counterpart, therefore we decided to develop the workflow around single z-slice segmentation. We calculated the cell volume of 228 single yeast cells using two methods: (a) segmentation of a specific z-slice of the phase contrast signal using YeaZ and (b) a mean z-projection of a fluorescent marker (mKate2 expressed from an additional *ACT1* promoter) using Cellpose. We found a strong correlation between the volume calculated with the two methods (Fig. 3), demonstrating the flexibility of the segmentation pipeline.

To highlight the possibility to analyse multiple model organisms, we applied the Cell-ACDC analysis pipeline to stem cells. Our results demonstrate that Cell-ACDC provides a tool for the unbiased and efficient analysis of fluorescent images of hematopoietic stem cells (HSCs) that is easy to use for researchers that do not have experience in using python. Using the automatic computation of key numerical features such as total fluorescence intensity and cell/nuclear volume included in Cell-ACDC, we showed that mTOR activity is largely constant with cell size, and that p38 activity is higher in smaller HSCs.

With the combination of sophisticated deep-learning algorithms and fast manual data correction, Cell-ACDC allows obtaining complete pedigrees over several cell cycles. As a proof of principle, we used this to quantify histone concentrations in budding yeast as a function of cell cycle progression, cell volume and replicative age. Our analysis revealed that the increase of cell volume during replicative ageing results in a decrease of histone concentrations.

Previously, developing a complete image analysis pipeline from existing tools required putting together different tools, such as ImageJ/Fiji [19], CellProfiler [22], and napari [56], and embedding state-of-the-art segmentation algorithms required extensive programming experience. Moreover, correction of segmentation and tracking errors as well as cell cycle annotation could in principle be performed with other tools such as YeaZ [5] GUI, DeepCell [31], PhyloCell [21] etc., but it required creating output data that can be loaded into these tools. Finally, calculating single-cell numerical features from fluorescent signals can be performed in ImageJ/Fiji or with custom code. Cell-ACDC aims at unifying all these steps in one single pipeline, where the data structure required is created only once as the first step. This is a great advantage not only because it speeds up the process but also because as the community adopts Cell-ACDC, it will foster collaboration and greatly reduce the complexity of sharing data between labs.

## Conclusions

### Future developments

We developed Cell-ACDC with a community-centred approach, by implementing suggestions from other research labs. We will keep this approach, and when adopted by a larger community, we envision a tool that can standardize live-cell imaging data processing and handling. Thanks to its modular backend, Cell-ACDC allows easy and fast implementation of image analysis models that will be developed in the future.

While initially developed for budding yeast, already in its current state Cell-ACDC can be used to obtain numerical features from images of any organism that can be segmented manually or using generalist models such as Cellpose.

In the current version of Cell-ACDC, full support for pedigree and cell-cycle analysis of symmetrically dividing cells is still missing. To address this, we plan to introduce a complementary automatic sister-pairing algorithm and division annotation in the future, which will allow lineage tree constructions and visualization.

Since image segmentation is often the first step in the image analysis pipeline, standardizing it will enable the development of more sophisticated downstream analysis methods (e.g. for sub-cellular feature extraction) that will be directly compatible with the output data generated by Cell-ACDC.

## Availability and requirements

Project name: Cell-ACDC

Project home page: https://github.com/SchmollerLab/Cell_ACDC

Operating system(s): Windows, macOS, Linux

Programming language: Python

Other requirements: Python 3.7, 3.8, or 3.8, Java 8 (optional)

Licence: BSD 3-Clause “New” or “Revised” License

Any restrictions to use by non-academis: None

## Materials and methods

### Software language and packages

The software is written in Python, freely available to all users. The code is open-source, and it is available at the GitHub repository https://github.com/SchmollerLab/Cell_ACDC. For automatic conversion of raw microscopy files into the required data structure, we embedded Java Runtime Environment (automatically downloaded) and *python-bioformats* to run the popular Bio-Formats [40] library directly from Python. Thanks to a GUI-based wizard, the user can automatically generate the required data structure. The GUI frontend is written using PyQt, a set of Python bindings for the Qt cross-platform C++ framework. Qt is a platform specifically designed for the development of fast and intuitive GUIs. To ensure a smooth user experience the images and the annotations are displayed using PyQtGraph, a Python library specifically designed for interactive and fast displaying of data. To easily add new models that will be developed in the future, we provide a drop-in approach, where any model placed in the “models” folder is automatically loaded. A GUI widget is automatically populated with the model parameters to easily adjust them. To ensure easy installation of Cell-ACDC, we provide ready to use virtual environments with the two most popular package installers, Anaconda and Pip. Finally, we provide a Quick Start Guide to start using Cell-ACDC as fast as possible and a User Manual (Additional file 5) that extensively documents every single function available. We describe the output data saved by Cell-ACDC in the Supporting Information.

### Live cell imaging

Fig. 2C shows strain KSY306-3 (*Mat a, his3::LexA-ER-AD-TF-HIS3 whi5::kanMX6-LexApr-WHI5-ADH1term-LEU2 exo84::EXO84-mCirine-ADH1term-cglaTRP1 cdc10::CDC10-mNeptune2.5-ADH1term-ADE2*) growing on SC media with 2% glycerol and 1% ethanol (SCGE) after pre-culture in SCGE with 20 nM β-estradiol. Data displayed in Figs. 1 and 2A, B, and D–G; and 5 was obtained from raw microscopy files included in our previous publication [53]. These figures show the strain KCY050-2. Data displayed in Figs. 2H and 3A was obtained from raw microscopy files included in our previous publication [57]. Specifically, Fig. 2H shows the strain KSY234-1; Fig. 3A shows strain KSY282-2. Briefly, live-cell time-lapse microscopy was performed using a Nikon Eclipse microscope equipped with a plan-apo λ 100×/1.45NA Ph3 oil immersion objective. Cells were imaged in a custom-made microfluidic device made of polydimethylsiloxane and a glass coverslip. A flow of 40 μl/min of synthetic complete liquid medium with 2% glucose was constantly applied at 30°C.

The diploid strain KCY050-2 carries endogenously tagged Htb1, while strain ASY020-1 was used as autofluorescence control [53]. Live-cell time-lapse microscopy was performed using a Zeiss LSM 800 microscope equipped with a plan-apochromat 40×/1.3NA oil immersion objective coupled to an Axiocam 506 camera. Note that Fig. 5B in essence reproduces the results for diploid cells in Fig. 1c of publication [53]. However, a different subset of cells from the raw data was analysed.

### Fluorescence staining of hematopoietic stem cells

Murine bone marrow (BM)-derived live G0/1 HSCs (Lin^-^, Sca1/Ly6^+^, CD117/cKit^+^, CD150/Slamf1^+^, CD48/Slamf2^-^, 7-ADD^-^) were isolated as described previously [46]. Briefly, BM was harvested by flushing the long bones. Red blood cells were lysed in ammonium-chloride-potassium (ACK) buffer and samples were washed in Iscove’s modified Dulbecco’s medium (IMDM) containing 2 % foetal bovine serum (FBS). BM cells were resuspended at 10 [6] cells/mL in pre-warmed IMEM supplemented with 2 % FBS and 6.6 μg/mL Hoechst-33342 (Thermo Fisher Scientific, #H3570). After 45 min of incubation at 37°C in a water-bath, cells were washed with cold IMEM with 2 % FBS and kept at 4°C. Lineage positive cells were depleted using a mouse lineage cell depletion kit and the following antibodies were used for staining: Rat monoclonal PE anti-mouse CD150, BD Biosciences, Cat#562651; RRID: AB_2737705; Rat monoclonal APC anti-mouse CD117, BD Biosciences, Cat#561074; RRID: AB_10563203, Armenian hamster monoclonal APC/Cy7 anti-mouse CD48, BioLegend, Cat#103431; RRID: AB_2561462, Rat monoclonal BV711 anti-mouse Ly-6A/E, BioLegend, Cat#108131; RRID: AB_2562241. Cells were sorted using an Aria cell sorter (Becton Dickinson).

For immunofluorescence analyses, Fisherbrand™ Superfrost™ Plus Microscope Slides were primed with 0.1 % polylysine for 5 min, washed with dH_2_O and air-dried. HSCs were distributed on slides and incubated for 1 h in a humidified chamber at RT. HSCs were fixed for 20 min at RT with freshly prepared 4% paraformaldehyde (PFA, pH 7.2) and then washed three times with PBS. HSCs were permeabilized for 20 min in 0.2 % Triton-X 100, washed three times with PBS, and blocked for 30 min using 10 % Donkey Serum (Sigma) in PBS. Cells were incubated with primary antibody in 10 % Donkey Serum in PBS overnight at 4 °C: Phospho-S6 Ribosomal Protein (Ser240/244) Rabbit mAb (Cell Signaling Technology, Cat# 5364; RRID:AB_10694233) or Phospho-p38 ( Thr180/Tyr182) (Cell Signaling Technology, Cat #9211, RRID: AB_331641). After HSCs were washed three times with PBS + 0.1 % Tween-20, the secondary antibody solution (1:500, goat anti-rabbit Alexa 488, Cell Signaling Technology, 4412S) was added for 1 h at RT in the dark in 10 % Donkey Serum in PBS. Coverslips were mounted with ProLong Gold Antifade Reagent with (Invitrogen, Molecular Probes) and imaged after 12 h. Control slides were not treated with primary antibody. Images were acquired using a DeltaVision Elite microscope (Applied Precision) platform (GE Healthcare Bio-Sciences) equipped with a CoolSNAP HQ2 camera (Roper), 250W Xenon lamps, SoftWoRx software (Applied Precision). Deconvolution was performed using SoftWoRx software with default settings. Cells that were 2.5 times larger than the mean were excluded from the analysis. To analyse HSCs of a specific size, the evaluated the 10 % smallest (XS-HSCs), the 10 % largest (XL-HSCs) and +/− 10 % HSCs of mean size (M-HSCs).

### Cell volume calculation

Cell volume is estimated from 2D segmentation masks as follows: (a) the object is aligned along its major axis, (b) the volume of each horizontal slice with 1 pixel height is calculated assuming rotational symmetry along the slice’s middle axis, and (c) volumes of the slices are added to obtain the final volume. We previously reported [57] that for budding yeast, this method well agrees with alternative methods, such as 3D reconstruction from z-stacks using confocal microscopy.

### Downstream analysis

For downstream analysis, we provide a notebook, written in python in the popular data science tool Jupyter Notebooks [55]. The user can select files to analyse by following a series of prompts and file dialogues, which also enables data pooling and comparison of subsets such as different strains or different conditions. The files selected are then iteratively loaded and geometric properties (e.g. area, solidity, elongation) are calculated using the package scikit-image [58]. Those quantities are complemented by additional parameters specific to time-lapse experiments, including cell age at frame *n*, growth rate, G1 and S/G2/M durations, as well as the first and last frames of cell appearance. In addition, signal amount and concentration for all available fluorescence channels are calculated. For this, the mean signal is corrected for background fluorescence by subtracting the median signal of all background pixels, which are determined as non-cell areas based on the cell segmentation masks. We define signal amount as corrected mean multiplied by the area of the segmented object (in pixels) and the signal concentration is obtained by dividing the amount by cell volume (calculated as described above). Note, that the fluorescence-related quantities can also be calculated directly in the GUI upon the user’s choice by selecting the option “Save additional metrics”.

We then perform two data aggregations using functions of the package pandas [59]. First, we connect the mother cell data with data of the respective buds and obtain single-cell time traces using the cell IDs. Second, we use generation number and cell cycle stage information to calculate cell-cycle-specific data.

Figure 3 was created using the output from Cell-ACDC without any pre-processing. Cell volumes and Alexa 488 concentrations were calculated as described above. In Fig. 5A, all individual cell cycle traces are aligned at bud emergence. To obtain the combined signal of mother cells and their buds, we summed the respective fluorescence signal amounts

### Continuous tracking

For the continuous tracking of single cells in the main GUI, we developed a cost-optimization routine to determine the optimal assignment of the segmented objects between two consecutive frames. First, a cost matrix **C** is computed: given a list **x** of object IDs [*x*_0_, *x*_1_…*x*_*n*1_] in frame *n* − 1, and a list **y** of [*y*_0_, *y*_1_…*y*_*n*2_] in frame *n*, each element *c*_*i*, *j*_ is equal to the intersection over area (*IoA*) score between *y*_*i*_ and *x*_*j*_. The *IoA* is calculated as the number of intersecting pixels between *y*_*i*_ and *x*_*j*_ divided by the area of *x*_*j*_. Next, any object with maximal *IoA* score less than 0.4 is considered a new object (e.g. a newly emerging bud), and receives a new ID. The remaining objects from frame *n* are assigned as follows: each unassigned object of list **y** is assigned to the object of list **x** with maximum *IoA* score unless the object from list **x** has a higher *IoA* with another object from list **y**. After having assigned objects from frame *n* to all objects from frame *n* − 1, the remaining objects are considered new and receive a new ID.

### Automatic separation of merged objects

Another algorithm embedded into Cell-ACDC is the automatic separation of merged objects. Since both Cellpose and YeaZ provide methods for separation, we developed our algorithm to provide an additional option for cases where Cellpose or YeaZ failed. The goal of the method is to separate the object along a restriction site. Firstly, the contour of the object is approximated to avoid spurious separation planes due to irregularities in the contour shape line. This is achieved with the OpenCV (computer-vision library for Python) [60] function approxPolyDP using 10% of the contour length as the epsilon parameter. Next, the convexity defects of the convex hull of the approximated contour are computed using the OpenCV function convexityDefects. Finally, if the number of detected defects is equal to two, then the object is separate along the line connecting the two convexity defects.

### Automatic mother-bud pairing

When the GUI is in “cell cycle analysis” mode, every new object appearing in the next frame is considered as a bud that needs to be assigned to a cell in G1 (if not already assigned in a previous visit of the frame). Firstly, the algorithm determines if there are enough cells in G1 for the new cells, and if not, a warning is triggered and the user can decide to automatically annotate that the history of these cells is not known (e.g. a cell appearing from outside of the field of view), or can annotate previous divisions of cells to increase the number of cells in G1 (if, for example, a division event was missed). After this checkpoint, the contour of each cell in frame *n* is computed. Then, given the lists **a**, **b** of the new cells and old cells in G1, respectively, a cost matrix **C** is calculated. Each *c*_*i*, *j*_ element is equal to the minimum Euclidean distance between the pixels of the *a*_*i*_ cell’s contour and the pixels of the *b*_*j*_ cell’s contour (in clustering referred to as “single link”). The optimal assignment is calculated using the minimum weight matching in bipartite graphs routine called the linear sum assignment problem (LSAP). To solve LSAP, we use a modified Jonker-Volgenant algorithm [42] implemented in the linear_sum_assignment function of the Python package SciPy [61]. This algorithm is one of the most popular variants of the “Hungarian algorithm”. One of the main strengths is that it is faster than the original implementation (*O*(*n*^3^) vs *O*(*n*^4^), with *n* being the number of objects to match). Currently, we solve the LSAP with information from a single frame. Including information from future and past frames might further increase the assignment accuracy.

### Automatic propagation of corrections to future and past frames

One of the most tedious and time-consuming processes is the correction of the same error when it appears in many consecutive frames. To speed-up this process we developed a series of routines to automatically propagate the correction to all the relevant future and past frames, when possible. Automatic propagation is triggered in the following situations: (a) mother-bud pairing correction, (b) cell division annotation and its correction, (c) tracking error correction, (d) object deletion, (e) editing a cell’s ID, (f) excluding a cell from analysis, and (g) annotating a dead cell. For situations c–g, the user can choose between applying the same correction/annotation to all future frames or simply repeat tracking for all the future frames. For situations a and b, the propagation is completely automatic. The correction of mother-bud pairing involves three cells: the bud, the wrong mother cell, and the correct mother cell. First, the correct mother cell must be a cell in G1, since the assumption is that each mother cell can have only one bud assigned to it. Furthermore, the correct mother must not have had any other bud assigned to it for all the frames in which the bud to be corrected is present. If the correct mother cell satisfies the eligibility criteria, once the user corrects the pairing, all the frames in which the annotation is wrong, are automatically corrected: the wrong mother cell goes back to the state it had before the bud was assigned to it, while the correct mother is assigned to the bud. Since correction is automatic to both past and future frames, it can be performed at any time point.

The correction of cell division annotation can be done on both a cell in G1 or a cell in S/G2/M. If the user clicks on a cell in S/G2/M (annotating division) at frame *n*, the automatic routine will annotate the division event at frame *n* for both mother and bud. Then, it will check if there are future frames that were previously annotated as cell in S/G2/M and will correct them accordingly. Otherwise, if the user clicks on a cell in G1 (undoing division annotation), the routine sets both the cell and the bud it had in the previous cycle back to S/G2/M for all the future (until the cell is in S/G2/M again or we reach the last visited frame) and past frames (until the mother cell is in S/G2/M again). Automatic propagation allows for annotating or undoing the annotation at any time point, which is particularly useful when toggling back and forth between frames is required for accurate cell division annotation.

### Benchmarking

Since the tracking algorithm is embedded into the main GUI, the key aspect is the execution speed (e.g. to spot subtle movements of a bud that indicate a cell division event). Therefore, we benchmarked it with a segmentation mask containing 99 cells to be tracked, and we calculated the average execution speed after 1000 runs. Our algorithm, on average, took about 45 ms, while the tracking algorithm embedded in the YeaZ model took about 260 ms. This is a considerable improvement that enhances the overall speed when navigating through frames in the main GUI. Finally, to allow the user to use the YeaZ tracking algorithm in real-time, we set out to improve YeaZ tracking speed. By optimizing the algorithm, we improved the computational speed by about 4-fold, from 260 ms down to about 60 ms.

Next, to benchmark the performance of Cell-ACDC we computed the widely used metric of the Multiple Object Tracking (MOT) challenge [62], the MOT accuracy (MOTA), defined as follows:

$$\mathrm{MOTA}=1-\frac{\sum_t\left(\mathrm{F}{\mathrm{N}}_t+\mathrm{F}{\mathrm{P}}_t+{\mathrm{IDSW}}_t\right)}{\sum_t{\mathrm{GT}}_t}$$

where *t* is the frame index, FN_*t*_ and FP_*t*_ the number of false negatives and false positives at frame *t*, IDSW is the number of identity switches at frame *t*, and GT_*t*_ is the number of ground-truth objects at frame *t*.

To cover multiple imaging conditions, we used images acquired with 4 different microscopes with more than 40,000 cells tracked. Details of the datasets are summarized in Table 2.

**Table 2:** ^a^ Dataset available at the URL http://yeast-image-toolkit.org/pmwiki.php

Test set	N. of unique cells	Total n. of cells	N. of videos	Frame count	Microscopes	Source
ACDC_TS	494	27,922	22	1710	Nikon Eclipse Ti-E, Zeiss LSM800	This manuscript
YIT_TS	960	15,662	10	250	Olympus PlanApo 1.4NA, Zeiss Observer Z1	Yeast Image Toolkit^a^

To compare to the Yeast Image Toolkit benchmark, along with MOTA, we also computed the *F*-score as reported on the benchmark website. We computed the number of correct links, *c*, the number of links in the prediction, *R*, and the number of links in the ground-truth, *G*. A link is defined as two consecutive points in a cell trajectory. Finally, the *F*-score is computed as follows:

$$F=\frac{2\ \left(p\mathrm{recision}\bullet \mathrm{recall}\right)}{\left(\mathrm{precision}+\mathrm{recall}\right)}$$

$$\mathrm{precision}=c/R$$

$$\mathrm{recall}=\frac{c}{G}$$

With these datasets, we benchmarked both YeaZ tracker and Cell-ACDC tracker under 3 different scenarios:

1. Uncorrected segmentation masks generated with the YeaZ model
2. Post-processed segmentation masks (from 1.) with automatic removal of false positives (more details below), where the optimal post-processing parameters were determined with a grid search
3. Segmentation masks corrected with Cell-ACDC (i.e., zero false positives and false negatives)

The post-processing consists of a computationally efficient method to remove false positives. Segmented objects are filtered with three parameters: minimum size, minimum solidity, and maximum elongation. Solidity is defined as the ratio of pixels in the object to pixels of the convex hull, while elongation is the ratio of the major to minor axis.

The results of the benchmark on the ACDC_TS dataset are summarized in Table 3.

**Table 3:** Multiple Object Tracking (MOT) metrics in 3 different scenarios (see main text). See main text for the MOTA formula

Scenario	Tracker	IDSW	FP	FN	MOTA mean	MOTA std.
1	Cell-ACDC	52	11,135	1482	0.498	0.910
	YeaZ	50	11,135	1482	0.498	0.909
2	Cell-ACDC	80	2838	1620	0.864	0.124
	YeaZ	57	2838	1620	0.865	0.001
3	Cell-ACDC	129	0	0	0.995	0.002
	YeaZ	11	0	0	0.999	0.001

*IDSW* identity switches, *FP* false positives, *FN* false negatives, *MOTA* MOT accuracy

The results indicate that while the Cell-ACDC tracker was developed by favouring computational speed to make it more suitable for real-time tracking, it scores very similar to the YeaZ tracker, indicating a minimal speed/accuracy trade-off.

Additionally, the post-processing introduced in Cell-ACDC dramatically improves accuracy of the trackers. Note that the best post-processing parameters were determined for each video, to show the full potential of the methods.

Notably, identity switches in the MOTA score are counted only when the switch happens for the first time, which means that it does not include information about the duration of the switch. For example, in scenario 2, using the YeaZ tracker we counted 57 identity switches, but the total duration of the switches is 2353 frames. This means that with a fully manual process, the user must manually edit 2353 cell IDs. Thanks to Cell-ACDC real-time tracking, only 57 edits are required, reducing number of manual corrections by more than 30-fold.

### Comparison to Yeast Image Toolkit dataset

To compare to the YIT benchmark, we created ground-truth segmentation masks of the YIT dataset using Cell-ACDC (ground-truth segmentation masks are not available on the YIT website). First, we segmented using either YeaZ or Cellpose (see Table 4), and then we visually inspected and corrected every video. We then performed tracking on these segmentation masks (same as scenario 3 in the MOTA benchmark), computed the F1 score and compared the results to the best algorithm tested on the YIT benchmark. The results are summarized in Table 4.

**Table 4:** F1-score for the tracking benchmark in comparison to the Yeast Image Toolkit benchmark

Test set	Segmentation model	Tracker	F1-score
TS1	YeaZ	Cell-ACDC	0.9983
		YeaZ	1.0000
		CellStar (best YIT)	0.9921
TS2	YeaZ	Cell-ACDC	1.0000
		YeaZ	1.0000
		Wood (best YIT)	1.0000
TS3	Cellpose	Cell-ACDC	0.9635
		YeaZ	0.9986
		CellStar (best YIT)	0.9852
TS4	Cellpose	Cell-ACDC	0.9448
		YeaZ	0.9979
		CellStar (best YIT)	0.9797
TS5	NE	NE	NE
TS6	Cellpose	Cell-ACDC	0.9975
		YeaZ	1.0000
		Wood (best YIT)	0.9698
TS7	Cellpose	Cell-ACDC	0.9013
		YeaZ	0.9105
		CellStar (best YIT)	0.9610
TS8	Cellpose	Cell-ACDC	0.9928
		YeaZ	0.9946
		Wood (best YIT)	0.9862
TS9	YeaZ	Cell-ACDC	0.9968
		YeaZ	0.9961
		Wood (best YIT)	1.0000
TS10	YeaZ	Cell-ACDC	1.0000
		YeaZ	1.0000
		Wood (best YIT)	1.0000

*NE* not evaluated

## Supplementary Information

**Additional file 1: Movie**. Video of a fully annotated position with cells disappearing due to suboptimal channel width.

**Additional file 2: Movie**. Visual help (rotating cell) in the main GUI. Cell 18 rotates at frame *n* + 1 resulting in a tracking error. Thanks to the annotations on the images, the user detects that cell 31 disappears, while a new cell 37 appears. To fix this, the user can manually assign ID 31 to cell 37. If the user does not see this and tries to continue to the next frame anyway, a warning message (pop-up window) will warn the user that cell 31 was lost and he/she can decide to continue or not.

**Additional file 3: Movie**. Automatic separation of merged mother-bud. After activating the “Automatic separation mode” with a button on the toolbar (or key shortcut), the user right-clicks on the merged objects to automatically separate them.

**Additional file 4: Figure S1**. Comparison between Cell-ACDC automatic separation algorithm and classic distance transform plus watershed.

**Additional file 5.** Cell-ACDC User manual. User manual with detailed explanation on how to use every module of Cell-ACDC.

**Additional file 6: Movie S4**. Cell cycle annotations example. When annotating cell cycle information, the user must keep an eye on two events: correctness of the automatic mother-pairing and division event. In this video, the user navigates through the frames and at a specific time-point the bud with ID=4 is automatically assigned to mother with ID=1. Next, when a sudden movement of bud with ID=4 is visible, the user clicks on the mother or bud to automatically annotate the division event.

## Data Availability

The source code is available on Zenodo [42] and on the GitHub repository at the following link: https://github.com/SchmollerLab/Cell_ACDC. All data generated or analysed during this study are included in this published article, its supplementary information files and publicly available repositories [63]. The repository also includes a smaller dataset for testing purposes.

## Abbreviations

ACDC: Analysis of the Cell Division Cycle
LSAP: Linear sum assignment problem
FCNN: Fully convolutional neural networks
HSCs: Hematopoietic stem cells
MAPK: Mitogen-activated protein kinase
