# Supplementary figures and images for: Segmentation, tracking and cell cycle analysis of live-cell imaging data with Cell-ACDC
Source: BMC Biol. 2022 Aug 5;20:174. doi: 10.1186/s12915-022-01372-6 (PMC9356409; doi:10.1186/s12915-022-01372-6)

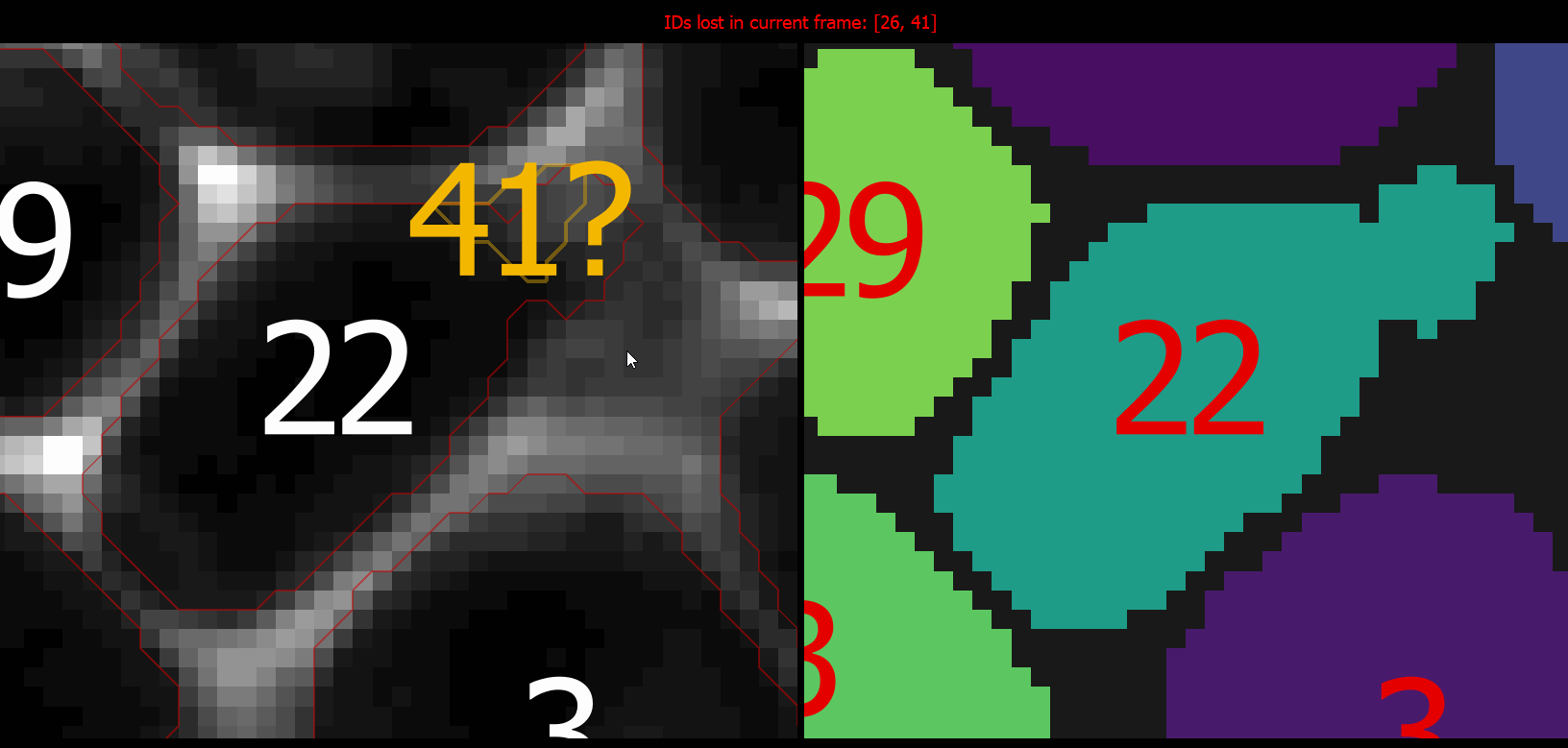

Supplement: Supplementary file 3 — Additional file 3: Movie. Automatic separation of merged mother-bud. After activating the “Automatic separation mode” with a button on the toolbar (or key shortcut), the user right-clicks on the merged objects to automatically separate them. [file 12915_2022_1372_MOESM3_ESM.gif]

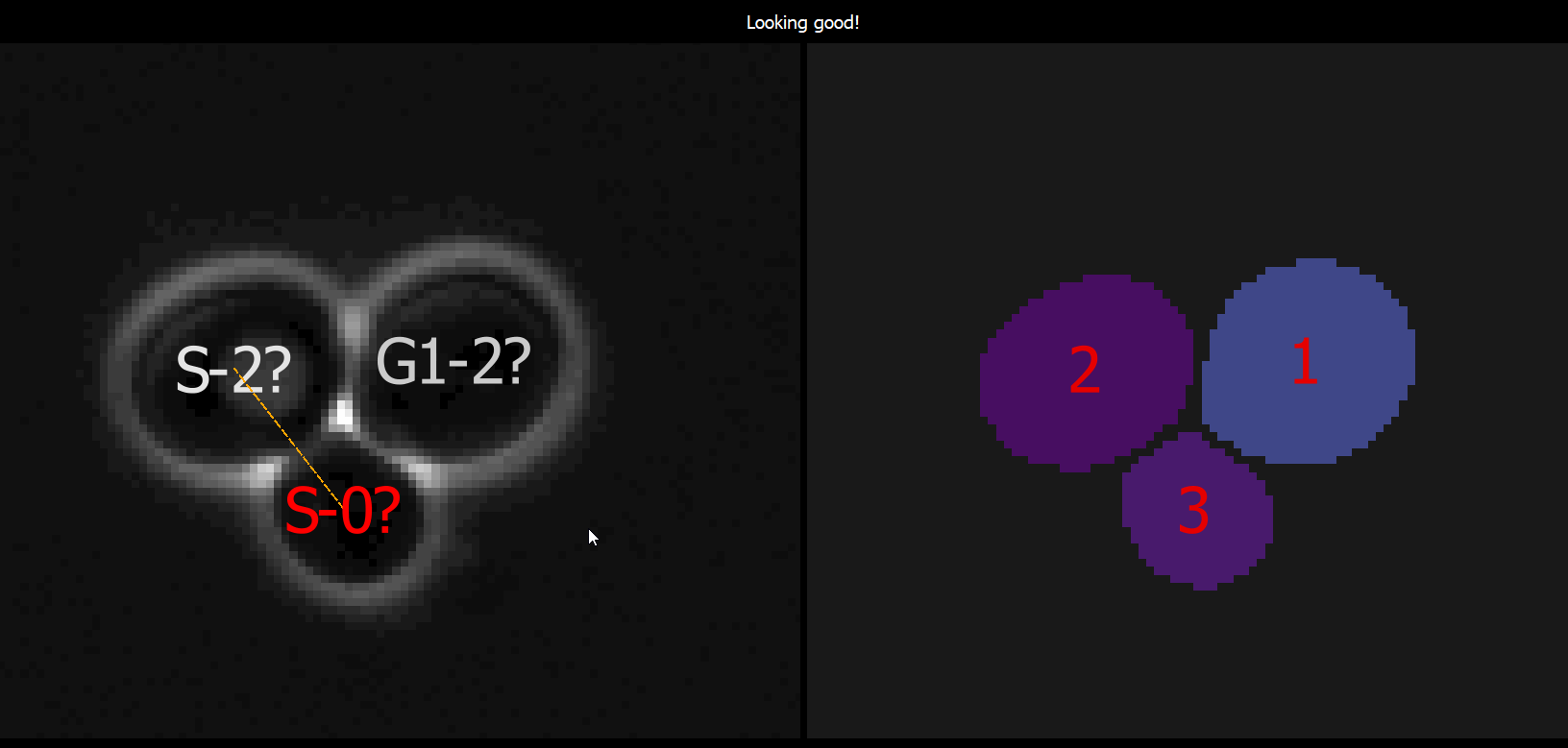

Supplement: Supplementary file 6 — Additional file 6: Movie S4. Cell cycle annotations example. When annotating cell cycle information, the user must keep an eye on two events: correctness of the automatic mother-pairing and division event. In this video, the user navigates through the frames and at a specific time-point the bud with ID=4 is automatically assigned to mother with ID=1. Next, when a sudden movement of bud with ID=4 is visible, the user clicks on the mother or bud to automatically annotate the division event. [file 12915_2022_1372_MOESM6_ESM.gif]
