# Supplementary material for: Segmentation, tracking and cell cycle analysis of live-cell imaging data with Cell-ACDC
Source: BMC Biol. 2022 Aug 5;20:174. doi: 10.1186/s12915-022-01372-6 (PMC9356409; doi:10.1186/s12915-022-01372-6)
Supplement: Supplementary file 4 — Additional file 4: Figure S1. Comparison between Cell-ACDC automatic separation algorithm and classic distance transform plus watershed. [file 12915_2022_1372_MOESM4_ESM.docx]

## Data saved by Cell-ACDC

All files saved by Cell-ACDC start with the same common basename, chosen by the user when converting the raw microscopy file into the required data structure (module “0. Create data structure from microscopy files(s)…”). The data is automatically organised into position folders, one position folder for each video/snapshot (more details can be found in the User Manual [here](https://github.com/SchmollerLab/Cell_ACDC/blob/main/UserManual/Cell-ACDC_User_Manual.pdf)).

The segmentation masks are saved in a compressed .npz file ending with “_segm.npz”. This file contains a Numpy array (Python’s library to handle numerical arrays) with the same shape as the segmented image. Each pixel in the mask has an integer value, 0 for the background and a specific ID for each pixel belonging to the same cell. The numerical features, such as mean, median, max, amount etc. of each channel’s signal, are saved in a tabular format into a file that ends with “_acdc_output.csv”. The CSV format allows for simple import into many other popular software and languages such as Excel, Matlab, R etc.

The user can create as many segmentation files as needed, for example one for each channel. Cell-ACDC will prompt for a name to append at the end of the file. The numerical features will also be saved in a file with the same name appended. For example, one could have a segmentation file that ends with “_segm_mCitrine.npz” with corresponding numerical features saved in the file ending with “_acdc_output_mCitrine.npz”.

To facilitate import of the segmentation file into other image analysis software, we provide utilities to convert the .npz file format into popular formats such as TIFF or HDF5 (.h5 extension).

Details about all other saved files (such as metadata or which z-slice/projection was used for segmentation, etc.) can be found in the User Manual (Additional file 5: section “Cell-ACDC output data”).


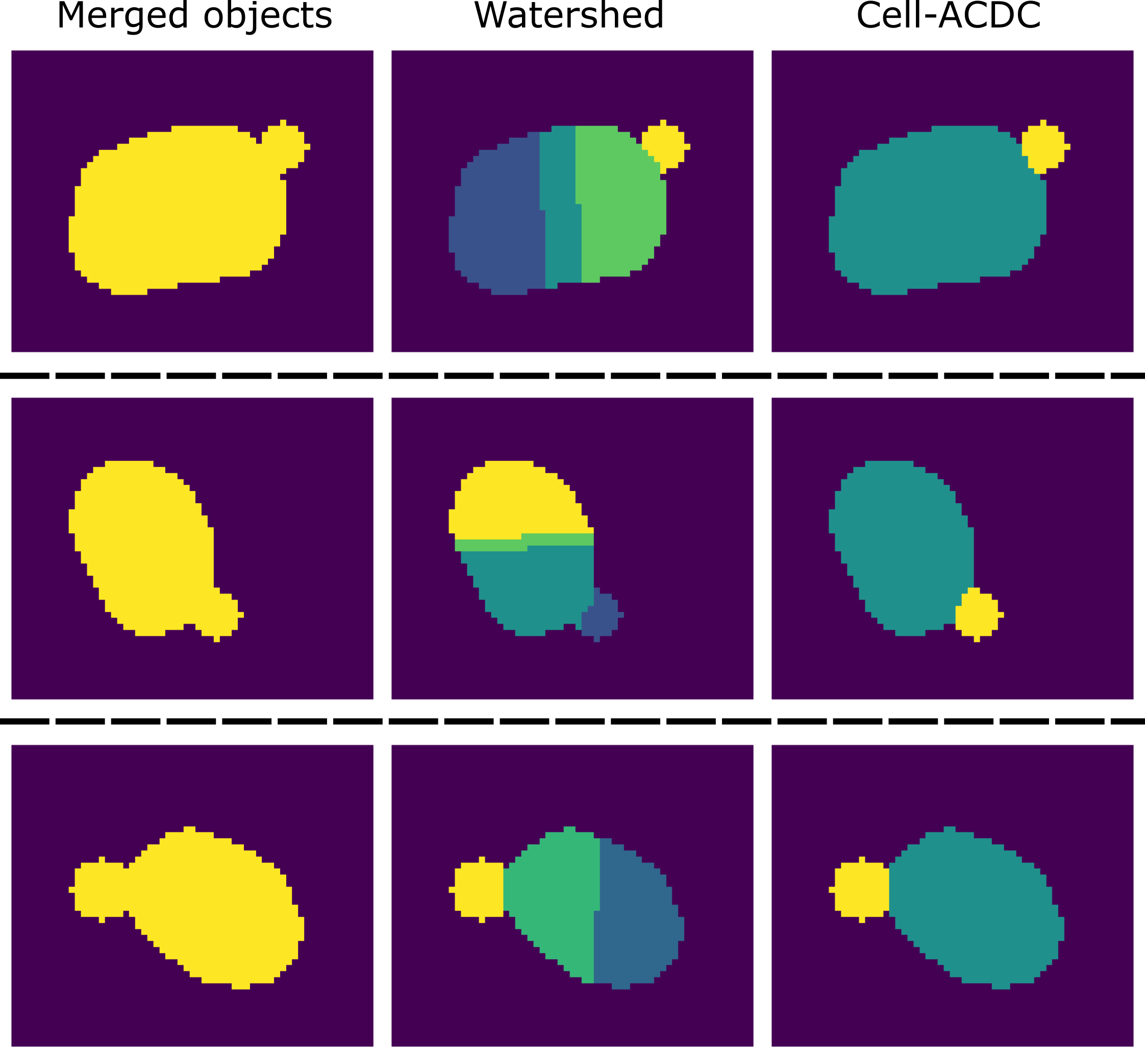


**Fig. S1** **–** **Comparison between Cell-ACDC automatic separation algorithm and classic distance transform plus watershed.** Three example cells, for which the separation method used by YeaZ fails. Note that the YeaZ splitting algorithm resulted in the objects not being separated, but for visualization purposes we show the results of the watershed separation. The YeaZ method attempts at removing wrong watershed lines by comparing them with the prediction of the neural network model. If the predictions that the lines are part of the cell are high enough, the lines are removed, and the objects are merged again. However, in these cases, the prediction at the mother-bud neck is high as well and the objects are merged again, resulting in bud connected to the mother cell. Cell-ACDC uses a different strategy. It uses a combination of convexity defects and contour approximation to determine the constriction site and it separates along that mother-bud neck. Note that this approach works only for two objects merged and separated by a constriction.
