# Supplementary material for: Segmentation, tracking and cell cycle analysis of live-cell imaging data with Cell-ACDC
Source: BMC Biol. 2022 Aug 5;20:174. doi: 10.1186/s12915-022-01372-6 (PMC9356409; doi:10.1186/s12915-022-01372-6)
Supplement: Supplementary file 5 — Additional file 5. Cell-ACDC User manual. User manual with detailed explanation on how to use every module of Cell-ACDC. [file 12915_2022_1372_MOESM5_ESM.pdf]

Cell-ACDC

A **GUI**-based framework for **segmentation**, **tracking** and **cell cycle annotations** of microscopy imaging data. It includes three of the most accurate deep learning methods, Cellpose, YeaZ, and YeastMate.

Cell-ACDC can load **2D**, **3D** (either single z-stacks or 2D images over time) and **4D** (3D z-stacks over time) images.

Written in Python 3 by Francesco Padovani and Benedikt Mairhoermann.

*Tested on Windows 10 64 bit, macOS , and Linux Mint 20.1

Installation

1. If you don't already have Python or Anaconda, download, and install Miniconda from here. ***IMPORTANT****: for Windows users makes sure you install the* ***64 bit version*** *of Miniconda*.

   We recommend using Anaconda even if you already have Python.
2. Follow the instructions below specific to your OS

Installing on Windows using conda

1. Open the Anaconda Prompt (you should be able to find it from the search bar)
2. If you already had Miniconda or Anaconda installed it might be a good idea to clean it first. Type the command `conda clean --all` and press "Enter".
3. Update conda by typing `conda update conda` and press "Enter".
4. **Create a conda environment** by typing `conda create -n acdc python=3.9` and press "Enter".
5. **Activate the environment** with the command `conda activate acdc`
   If activation was successful, you should see the writing `(acdc)` on the left of the path where you type (see red circle in the screenshot below).

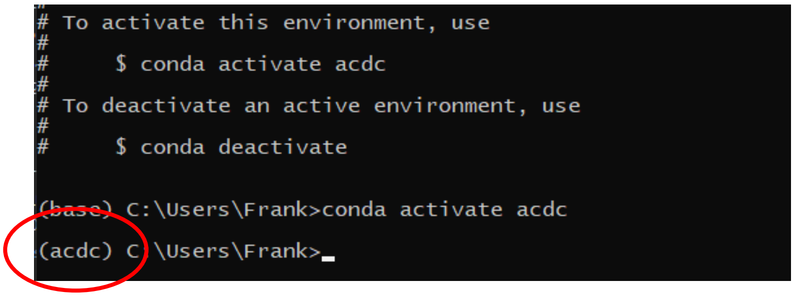

6. **Install Cell-ACDC** by typing `pip install cellacdc` (make sure to have internet connection) and press "Enter". Keep an eye on the terminal in case of any error.
7. To launch Cell-ACDC activate the environment (if not already active) then type the command `acdc` and press "Enter".

NOTE: If you had an error, you could try installing using pip (see instructions below) or open an issue here.

Installing on Windows using pip

1. Download and install Python 3.8 or Python 3.9.7 from here. **Make sure to check the option** Add Python 3.9 to PATH and then install with default options.

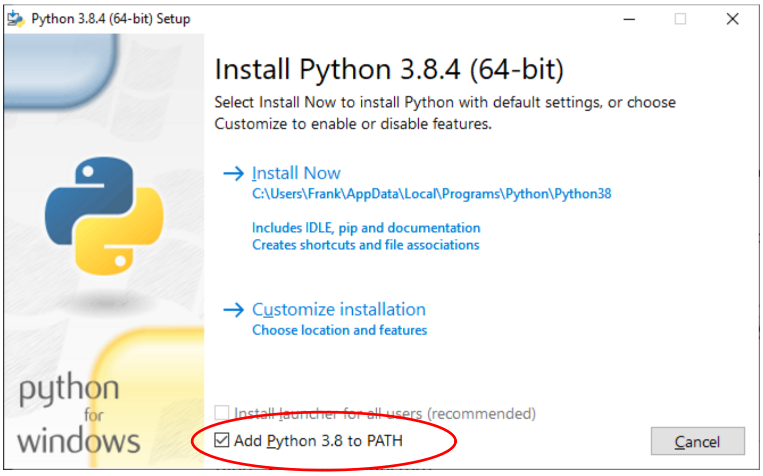

2. Open a terminal (either a Command Prompt or PowerShell, you can find both from the search bar. I recommend installing Windows Terminal and use the PowerShell)

3. Create a folder for the virtual environment and then navigate to that folder (in this example it is `C:\Users\Frank\venvs`) by typing `cd “C:\Users\Frank\venvs”` Press *Enter* to confirm.

4. Now type the following commands **one at the time** (press “Enter” after each command and type “Y” when requested):

   `py -m pip install --upgrade pip`

   `py -m venv acdc`

   `.\acdc\Scripts\activate` ← *This activates the environment*

   `py -m pip install cellacdc`

   If activation was successful, you should see the writing `(acdc)` on the left of the path where you type (see red circle in the screenshot below).

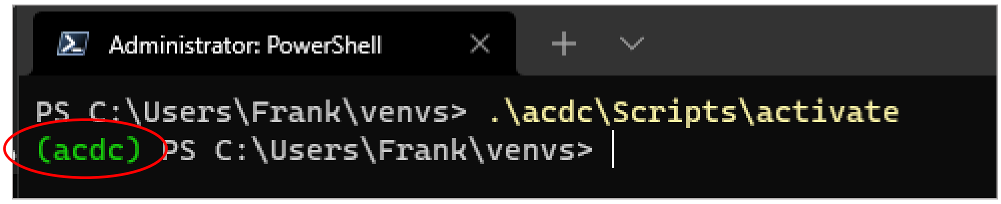

5. To launch Cell-ACDC activate the environment (if not already active) then type the command `acdc` and press “Enter”.

Installing on macOS/Linux using conda

1. Open a **Terminal** (Click the Launchpad icon in the Dock, type “Terminal” in the search field, then click Terminal)
2. If you already had Miniconda or Anaconda installed it might be a good idea to clean it first. Type the command `conda clean --all` and press “Enter”.
3. Update conda by typing `conda update conda` and press “Enter”.
4. **Create a conda environment** by typing `conda create -n acdc python=3.9` and press “Enter”.
5. **Activate the environment** with the command `conda activate acdc`
6. **Install Cell-ACDC** by typing `pip install cellacdc` (make sure to have internet connection) and press “Enter”. Keep an eye on the terminal in case of any error.
7. To launch Cell-ACDC activate the environment (if not already active) then type the command `acdc` and press “Enter”.

   NOTE: If you had an error, you could try installing using pip (see instructions below) or open an issue here.

Installing on macOS/Linux using pip

1. Download and install Python 3.9.7 from here. Install with default options.

2. Create a folder for the virtual environment and then navigate to that folder (in this example it is `/Users/Frank/venvs`) by typing `cd “/Users/Frank/venvs”` Press *Enter* to confirm.

3. Open a **Terminal** (Click the Launchpad icon in the Dock, type “Terminal” in the search field, then click Terminal)

4. Now type the following commands **one at the time** (press “Enter” after each command and type “Y” when requested):

   `python3 -m pip install --user --upgrade pip`

   `python3 -m venv env`

   `source env/bin/activate` ← *This activates the environment*

   `python3 -m pip install cellacdc`

5. If activation was successful, you should see the writing `(acdc)` on the left of the path where you type.

6. To launch Cell-ACDC **activate the environment** (if not already active) then type the command `acdc` and press “Enter”.

   NOTE: If you had an error, you could try installing using pip (see instructions below) or open an issue here.

First steps

Starting the main launcher

1. Open a terminal:
    - Windows: **Anaconda Prompt** if you installed with **conda**
    - Windows: Command Prompt or **PowerShell** if you installed with **pip**
    - Unix/maxOS: **Terminal**
2. If you installed with pip first navigate to the folder where you created the virtual env (in the examples it was `/Users/Frank/venvs` on macOS and `C:\Users\Frank\venvs` on Windows
3. **Activate** the environment:
    - Conda: `conda activate acdc`
    - pip on Windows: `.\acdc\Scripts\activate`
    - pip on Unix/macOS: `source env/acdc/activate`
4. Run the main launcher with the command `acdc` or `cellacdc`
5. The first time, it will take 1 or 2 minutes to launch. The next times it will be faster. Once launched, you should get the following Welcome Guide window.

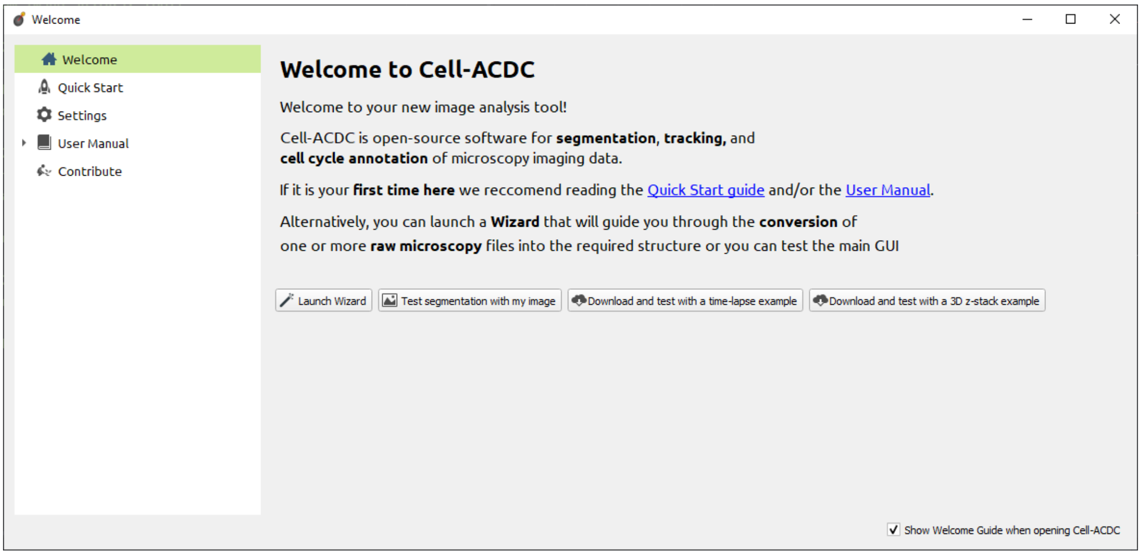

Create required data structure from microscopy file(s)

To load a microscopy file, Cell-ACDC uses the java library Bio-Formats and the Python library python-bioformats. The python-bioformats library was developed for CellProfiler and it is **embedded** into Cell-ACDC. It essentially allows you to run the java code from Bio-Formats from Python. Have a look here for a list of supported file formats.

To load a microscopy file into the Cell-ACDC pipeline, we first have to **convert it into a specific data structure**. We included a module that allows you to **automatically create the required data structure**. However, if it fails, you can create it manually with ImageJ/Fiji. Read the section of this manual called "Manually create data structure from microscopy file(s)".

1. From the main launcher (could be behind Welcome Guide window) click on the "Create data structure from microscopy file(s)..." button and follow the instructions of the Wizard.

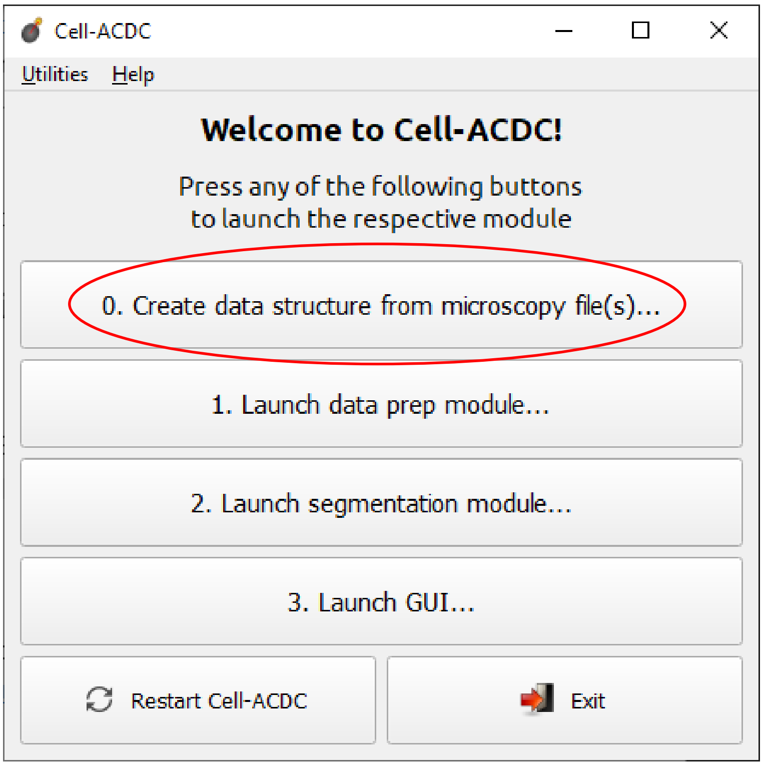

2. Once the creation of the data structure is finished, you are ready to start using your new labelling tool! The easiest way to start is from the Quick Start section of this User Manual.

Create data structure using Fiji Macros

1. If you don't have Fiji, download and install it from [here]

2. Download the Fiji Macros from [here]

3. Open the Fiji app. Go to File → Open… and then go to the folder where you downloaded the Fiji Macor.

    a. If you have a **single microscopy file for each experiment** containing multiple positions (with or without multiple channels) open the `single_file.ijm` file.

    b. If you have **multiple microscopy files for each experiment**, (one file for each position/series) open the `multiple_files.ijm` file. In this case, you need to put all the microscopy files from the same experiment into an empty folder.

4. Before running the macro, you have to **edit the channels**. At about line 3 modify the `channels` variable by writing a name for each channel in your file. They **MUST be in the exact same order they are in the original microscopy file**. If you don't know the order, open the file in Fiji first and check the order of the channels there.

5. Run the script with the `Run` button. The script will ask you to select a folder containing the microscopy files (`multiple_files.ijm`) or a microscopy file (`single_file.ijm`). Select the folder/file and wait. You can see the progress in the Log Window. The script will silently open the microscopy files and save each Position (if the file contains multiple positions) into a separate folder.

If successful, you should now have a **new folder for each position** called Position_1, Position_2 etc. Inside each Position folder you should have a folder called **Images**. Inside Images you now have **one .tif file for each channel**.

Check that each channel name (appended at the end of the .tif file) corresponds to the actual data contained in the .tif file. If it doesn't correspond, delete all the created folders, and repeat the process with the **right order of channel names** in the variable called `channels` (point 3.).

Manually create data structure from microscopy file(s)

7. If you don't have Fiji, download and install it from here
8. Open the Fiji app and launch the Bio-Formats importer from the menu "Plugins → Bio-Formats → Bio-Formats Importer" and select your microscopy file (one at the time).
9. Check the options "Use virtual stack" and "Split channels" as in the screenshot below

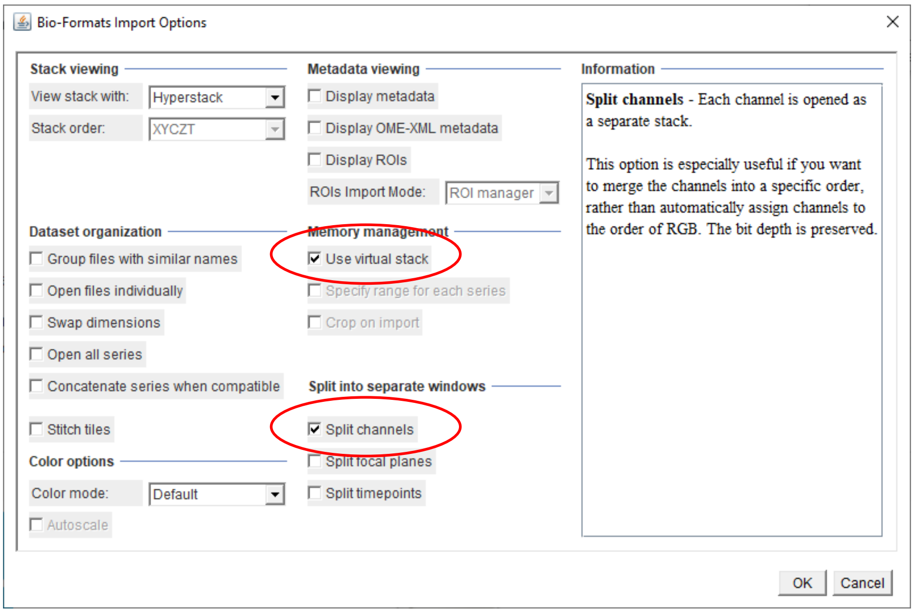

10. If you have multiple positions (series) in the file you opened, you will be asked to select a position. We recommend opening one position at the time, to avoid memory issues.
11. You should now have one image window for each channel you had in the file. Select the window with the image data from the first channel (the window name should be something like "filename … C=0"), then "File → Save as… → Tiff"
12. As a filename we recommend calling it with the same name of the original microscopy file (if it is not too long) **WITHOUT the extension** plus something like "_s01_channel0.tif", where instead of "channel0" you can write whatever you like (e.g., DAPI or GFP etc.) and "s01" is for first position. So, for example the phase contrast channel of a .czi (Zeiss microscope) file called `ASY15-1_15nM-01.czi` can be save as `ASY15-1_15nM-01_s01_phase_contr.tif`
13. Save the .tif file to a path called "/Position_1/Images".
14. Repeat 2-7 for all the other positions.
15. In the end you should have the following folder structure:

```
Experiment_folder
  ├─► Position_1
  │     └─►Images
  │          ├─► basename_s01_channel0.tif
  │          ├─► basename_s01_channel1.tif
  │          ├─► …
  │          └─► basename_s01_channeln.tif
  ├─► Position_2
  │     └─►Images
  │          ├─► basename_s02_channel0.tif
  │          ├─► basename_s02_channel1.tif
  │          ├─► …
  │          └─► basename_s02_channeln.tif
  │   …
  └─► Position_n
        └─►Images
             ├─► basename_s0n_channel0.tif
             ├─► basename_s0n_channel1.tif
             ├─► …
             └─► basename_s0n_channeln.tif
```

16. Once the creation of the data structure is finished, you are ready to start using your new labelling tool! The easiest way to start is from the Quick Start section of this User Manual.

Quick Start

Cell-ACDC is composed of **three main modules**:

- **Data prep**: align time-lapse data, crop, and select a z-slice or z-projection and a ROI for segmentation. More details…
- **Segmentation**: to automatically segment multiple experiments and multiple positions with the embedded deep learning models (YeaZ for yeast cells and Cellpose for various model organisms). More details…
- **Main GUI**: to visualize segmentation masks, correct segmentation and tracking errors, and cell cycle annotations. More details…

The easiest way to start is to **open the main GUI**.

Next, if you already created the data structure (see Load microscopy file section) you can click on the "Open Folder" button on the toolbar, otherwise you can go to "File →Open image/video file…". To start the main GUI, click the "Launch GUI…" button on the main launcher.

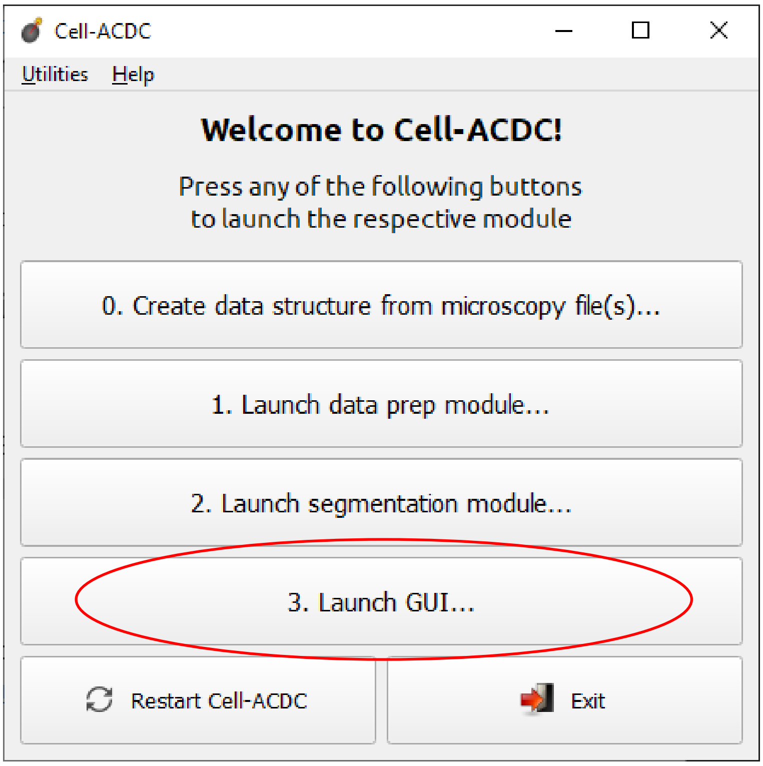

Data Prep module

To use the data prep module, you need to **first create the required data structure.** See this section.

Use the **Data Prep** module if you need to do one of the following **tasks**:

a) Select a **z-slice** or **z-projection** for segmentation of 3D z-stacks.

b) **Align frames** of time-lapse microscopy data (RECOMMENDED, it is revertible).

c) Calculate **background metrics** (median, mean etc.) from one or more **rectangular areas**. The median will be used later for background subtraction. The areas are movable and resizable.

d) Select a region of interest (**ROI**) for segmentation.

e) **Crop** images to reduce memory usage (RECOMMENDED, if possible).

Loading data

1. **Launch** the data prep module, click on the "1. Launch data prep module…" button on the main launcher.
2. Click on the "**Open Folder**" button on the toolbar.

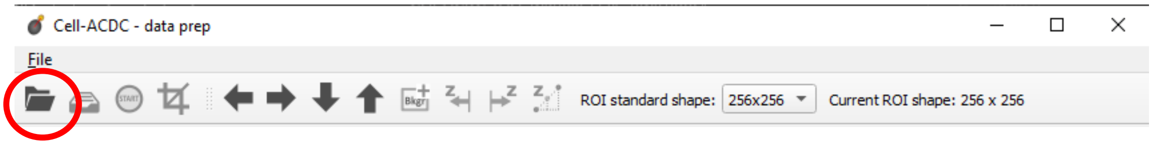

3. Select a specific Position folder or the entire experiment folder.

4. Follow the instructions in the pop-up windows. Make sure to enter the **correct metadata.**

   NOTE: For time-lapse microscopy you can load only one position at a time. Select multiple positions only if you have single 3D z-stacks or single 2D images.

Usage

1. If your data does not contain 3D z-stacks go to point 2. Otherwise, you can visualize the **z-slices** with the **scrollbar** below the image or choose a **z-projection** method with the selector on the right side of the scrollbar.

   Every time you change the visualization method, the system will save it. It will then assume that the **last visualization is the preferred one** and it will be used for **segmentation**.

   For **time-lapse data** you have **additional buttons** to help with the selection. Go to the section "Additional functions" for details about their functionality.

2. If you do not need to select a ROI, crop, align or calculate background metrics you can close the window. Otherwise press the “**Start**” button on the toolbar and follow the instructions on the pop-up windows.

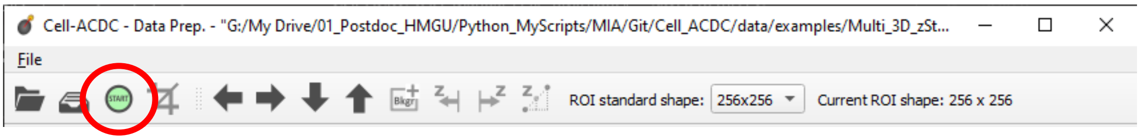

3. The GUI now will be **unresponsive** until the process terminates, so do not close it. You can follow **progress** in the **terminal**. Once it finishes, a **red rectangle** will appear, along with a grey rectangle (see screenshot below). If you do not need to select a **ROI,** calculate **background metrics,** or crop you can close the window now, otherwise go to the next point.

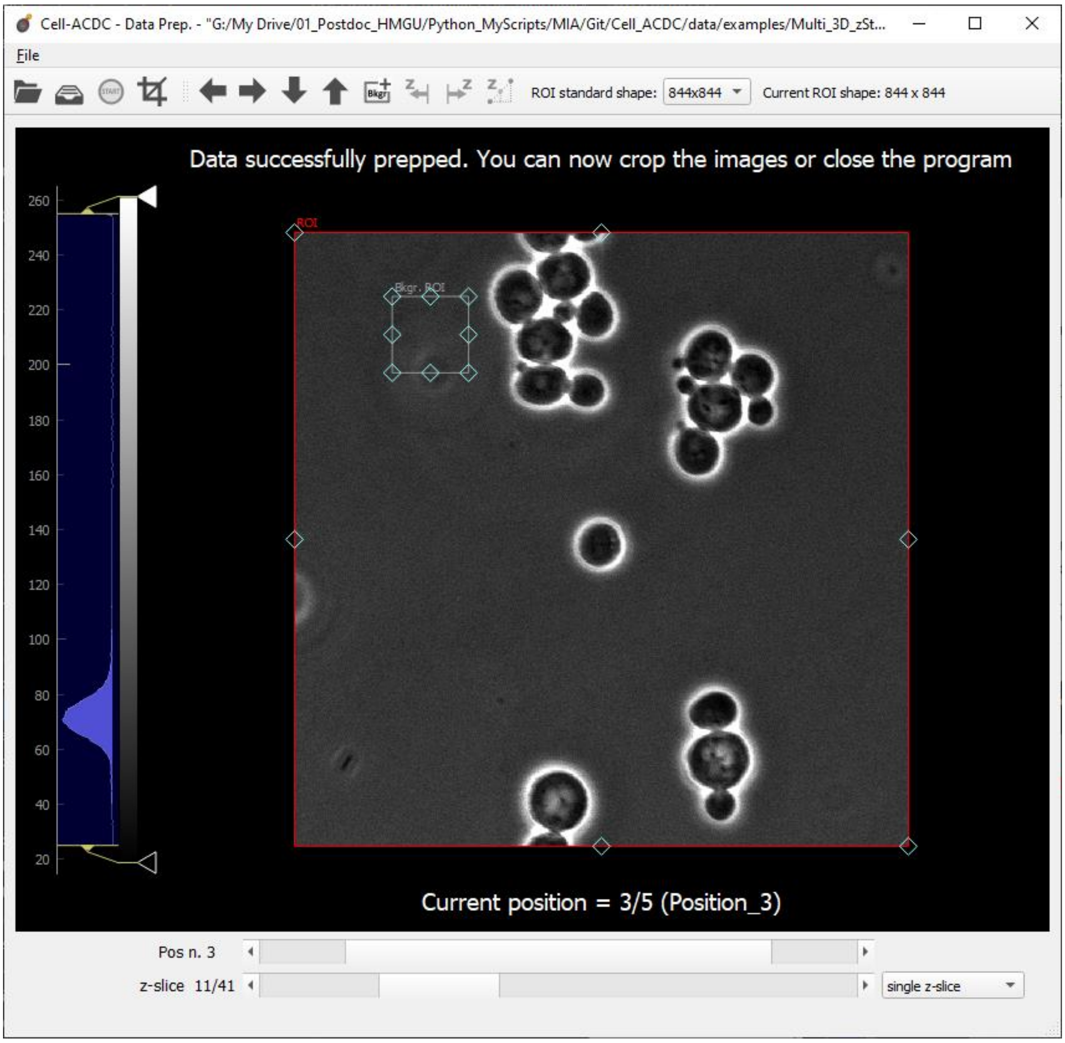

4. The **red ROI** is used for either **cropping** or saving the coordinates where to compute **segmentation**. The **grey ROI** (Bkgr. ROI) is used to calculate **background metrics** from that area (median, mean, quantiles etc.). You can add more background ROIs with the “Add ROI where to calculate background intensity” (Bkgr) button on the toolbar.

5. **Resize** and **move** the ROIs until you are happy with their position and size, click on the **green tick** button on the toolbar, then follow the instructions in the pop-up windows. The GUI will be **unresponsive** until the process terminates, so **do not close it**. You can check progress in the terminal.

Additional functions

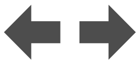 Go to **previous/next** position or frame (time-point).

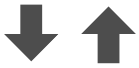 Go **10** positions or frames **backward/forward.**

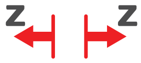 Use the same **z-slice** from current frame to all past/future frames.

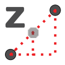 Use **linearly interpolated z-slices** from first frame to current frame.

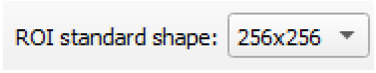

Select one of the **standard shapes** for the red ROI.

Segmentation module

The segmentation module is used for **automatically segmenting multiple experiments and multiple positions** in one session.

To use the segmentation module, you need to **first create the required data structure.** See this section.

NOTE: if you are just testing, you can also segment in the main GUI. Use this module when you need to segment many experiments and/or many positions.

Usage

To use this module, simply follow the **instructions** in the **pop-up windows**. To **launch** the module, click on the "2. Launch segmentation module…" button on the main launcher.

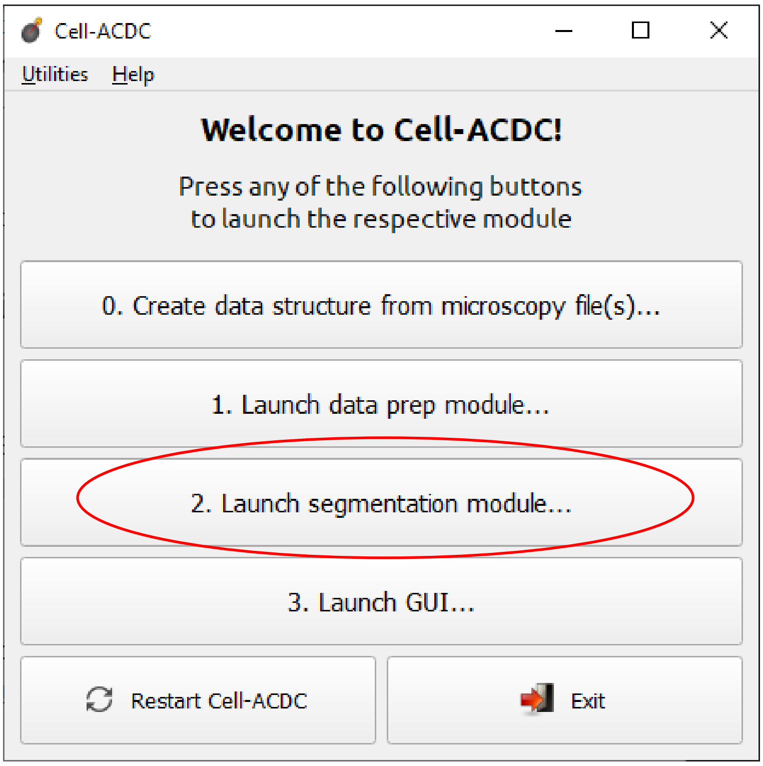

Main GUI

The main GUI is the actual **core of Cell-ACDC**. It serves multiple purposes:

a) **Test** which **segmentation method** works best for your dataset.

b) **Correct** segmentation and tracking errors.

c) Cell cycle **annotations.**

As for all the other modules, you can load **2D**, **3D** (either single z-stacks or 2D images over time) and **4D** (3D z-stacks over time) images or videos.

Loading data

1. **Launch** the GUI module by clicking on the `3. Launch GUI...` button on the main launcher.

2. Depending on the data structure, do one of the following actions:

   - If you already created the **data structure** following the instructions in this section (recommended) then click on the Open Folder button.

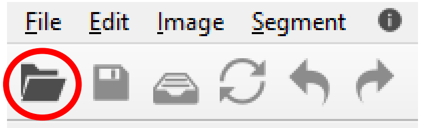

   - If you have a single image (.tif, .png, .jpg, etc.) or video (.mov, .avi) go to `File → Open image/video file...`

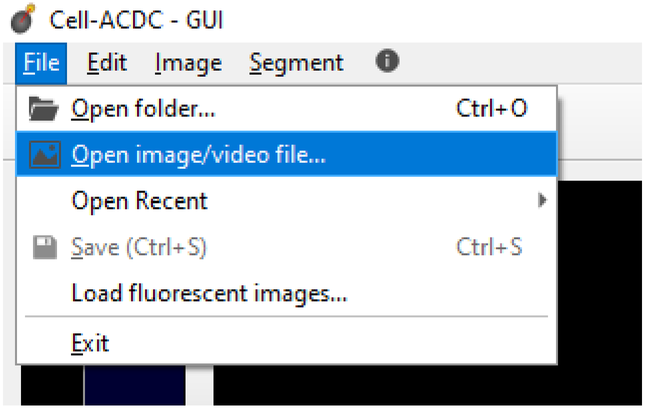

3. Select a specific file, a Position folder, or the entire experiment folder.

4. Follow the instructions in the pop-up windows. Make sure to enter the **correct metadata.**

*NOTE: if you load a **single image** or **video** file **without the required data structure,** the Cell-ACDC output will be saved in a sub-folder called* `<timestamp>_acdc_output`

Usage with time-lapse data

For **time-lapse data**, you can load one position (one video) at a time. With this data, the GUI has **three modes** that can be toggled from the selector on the toolbar:

a) Viewer mode (default mode, used only for visualisation).
b) Cell cycle analysis mode.
c) Segmentation and tracking mode.

The **main idea** is that when you visit a frame for the first time, some automatic functions are triggered: **tracking** in Segmentation and tracking mode, **mother-bud pairing** in Cell cycle analysis mode.

These functions are **not triggered** when you visualize a frame that you **already visited before**. You can always call any function manually (see this, this, or this section of the manual).

Give a **quick read** to

Tips and tricks and then **start using the GUI**.

If you are unsure about the function of any item in the GUI, see this, this, or this section of the manual.

Usage with snapshot data (no time-lapse)

For **snapshot data**, you can load **multiple positions** at the same time. When prompted, simply click on multiple selection button, and then select the positions with `Ctrl+click` for selecting specific positions, or `Shift+click` to select a range, or `Ctrl+A` to select all.

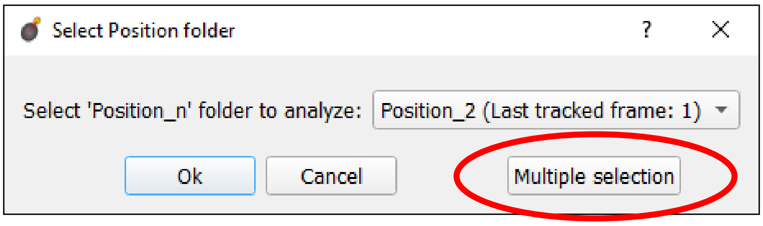

Once loaded, you can **navigate** through positions with **left and right arrow** or with the Position **scrollbar** below the left image.

Give a **quick read** to Tips and Tricks and then **start using the GUI**.

If you are unsure about the function of any item in the GUI, see this, this, or this section of the manual.

Tips and tricks

- Most of the **functions** are available from the **toolbar** on the top of the window:

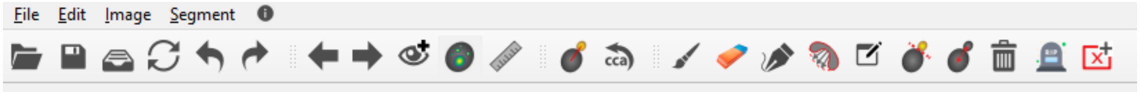

- Activate the function with a ***SINGLE-click*** on the button
- When you **hover a button** with the mouse cursor you get a **tool tip** on how to use that function:

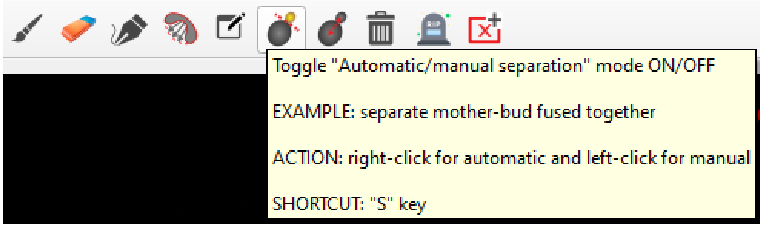

- The **tool tip** will tell you whether you need ***RIGHT-click*** or ***LEFT-click*** for that function
- Functions **NOT** present on the **toolbar**:
  - `Middle-click` (scrolling wheel) on Windows, `Cmd+Click` on macOS → delete the segmented object you click on
  - `H` key → automatic zoom on the segmented objects
  - `Double press H` key → zoom out
  - `Ctrl+P` → visualize cell cycle annotations in a table
  - `Ctrl+L` key → relabel object IDs sequentially (1,2,3...etc)
  - `Spacebar` key → hide/show contours or segmentation masks on left image
  - `Ctrl+F` key → search and highlight specific object ID
  - `Alt+Click+Drag` → pan/move image

- To **test** the available **segmentation models,** use the Segment menu.
- To **navigate frames** (time-lapse data) or **positions** (snapshots data), use the ←→ **arrows** on the keyboard.
- To visualize the frames of time-lapse data in a second window click on the Slideshow button on the toolbar: 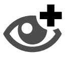
- **Personalize** settings such as **Font Size**, **overlay colour** and **text's colour** from the Edit menu.

Functions activated from the toolbar

File toolbar

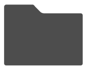

Open folder: used to **automatically load** single or multiple positions from the standard data structure. See this section for details on how to create the required data structure**.**

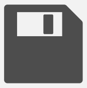

Save: used to save **all data** (segmentation mask, cell cycle annotations and metrics calculated from the loaded fluorescent images).

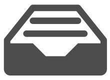

Show in Explorer/Finder: open the loaded folder into your Explorer/Finder.

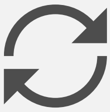

Reload segmentation file: used to reload the segmentation labels from the hard drive. Use it if you want to go back to the saved state.

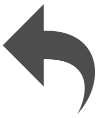

Undo (*Ctrl+Z*): almost all the performed actions are undoable. Currently you can undo up to 5 actions in the past.

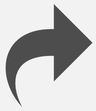

Redo (*Ctrl+Y*): repeat an undone action.

Visualize toolbar

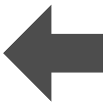

Go to previous frame (time-lapse data) or Position (snapshot data).
SHORTCUT: Left arrow on the keyboard

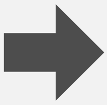

Go to next frame (time-lapse data) or Position (snapshot data).
SHORTCUT: Right arrow on the keyboard

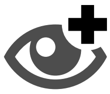

Open the images in a **second window.** This window will have no annotations, which means it is the fastest way possible to visualize the frames. Handy in many situations.

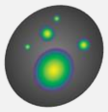

Overlay a second signal: when you press this button for the first time, you will be asked to choose which signal to overlay. The next times it is used to toggle **overlay on/off**.

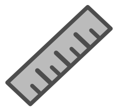

Ruler: draw a line with the left button to **measure the distance** between two points (*pixels* and *µm*). Distance will be displayed in the bottom-left corner of the window.

Edit toolbar

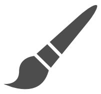

Brush (*left-click motion*)**:** used to modify a segmented object or paint a new object with a circular brush. Increase/decrease the size of the brush with up/down arrows on the keyboard. *SHORTCUT: B key*

Two **modes**:

- To **draw a new object** start painting on the background (brush cursor is white).
- **Modify an object** by starting to paint from the object (brush cursors takes the color of the object you are about to modify)

NOTE: The brush will add the new object UNDER existing objects unless you first press the B key twice. The brush button will then turn red and new objects will be added ABOVE existing objects. Restore default behaviour by pressing B twice again.

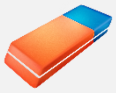

Eraser (*right-click motion*): used to erase parts of a segmented object with a circular cursor. *SHORTCUT: X key*

Two **modes**:

- Start erasing from the background to erase **all the objects** you will pass over (eraser cursor is red).
- Start erasing from a specific object to erase ONLY that object (eraser cursor keeps the colour of the object you start erasing from).

NOTE: To enforce erasing any object you pass over, even if you start from a specific one, press the X key twice. The eraser button will then turn RED. Restore default behaviour by pressing X twice again.

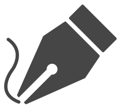

Curvature tool: used to draw new objects by drawing a spline with multiple anchor points. *SHORTCUT: C key*

Two **modes**:

- Consecutive **left-clicks** to **manually** draw an object with a spline.
- **Right-click** with a drawing motion to **automatically** follow an intensity line.

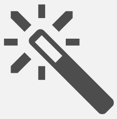

Magic wand: draw new object by clicking on an intensity pixel. Adjacent pixels that have intensity values +- tolerance will be automatically part of the object.
SHORTCUT: W key

Usage: first activate the button (left-click or shortcut) and then *LEFT-click* on the left image.

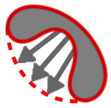

Hull contour: replace object with its hull contour image. Useful to **fill holes** and **cracks**.
SHORTCUT: F key

Usage: first *activate the button* (left-click or shortcut) and then *RIGHT-click* on the requested object.

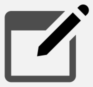

Edit ID: replace the ID of an object with a manually inserted one. *SHORTCUT: N key*

Usage: first *activate the button* (left-click or shortcut) and then *RIGHT-click* on the requested object.

Separate objects: used to separate merged objects. *SHORTCUT: S key*

Usage: first *activate the button* (left-click or shortcut) and then *RIGHT-click* on the requested object to first attempt **automatic separation** (works well with two objects separated by a constriction) or *LEFT-click* to go straight to **manual separation**.

When **manual separation** is triggered, a window with only the object you clicked one will appear (see screenshot below). To separate the object along a curved line, you need **three *RIGHT clicks***:

1. First *RIGHT-click* where to **start** a straight line.
2. Second *RIGHT-click* where to **end** the straight line.
3. Third *RIGHT-click* to **set the curvature** of the separating curve.

The object will then be separated along the drawn line.

*NOTE: to **help** with deciding where to draw the line you can **overlay** the intensity signal with the overlay button*

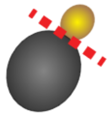

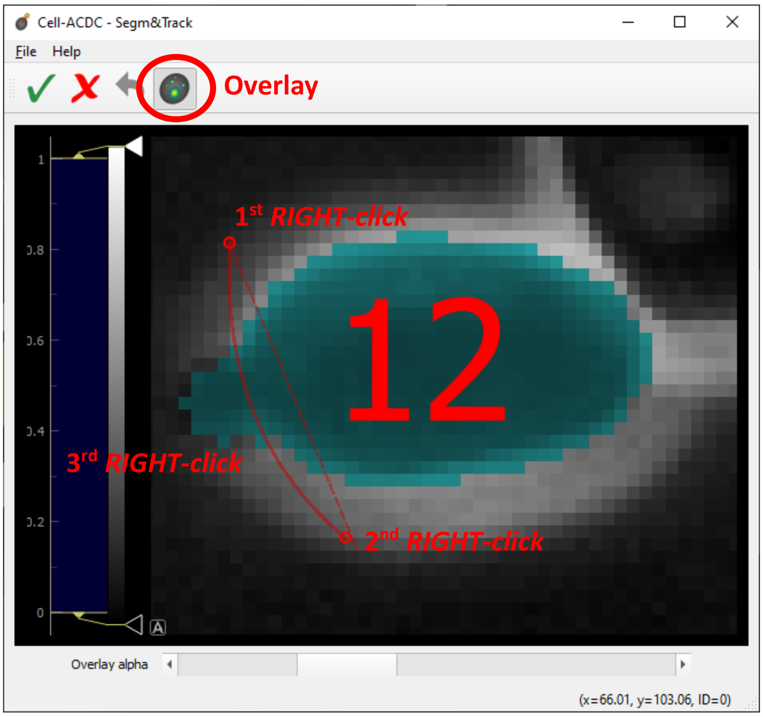

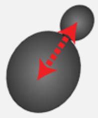

Merge objects. *SHORTCUT: M key*

Usage: first *activate the button* (left-click or shortcut) and then *drag-and-drop* motion with *RIGHT-button* between the two objects.

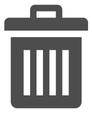

Annotate cell as "Excluded from the analysis": the column called `is_cell_excluded` (on the saved data, see this section) will have a TRUE for the clicked object from current frame to the end. *SHORTCUT: R key*

Usage: first *activate the button* (left-click or shortcut) and then *RIGHT-click* on the requested object. Repeat to undo from that frame onwards.

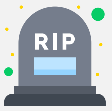

Annotate cell as "Dead": the column called `is_cell_dead` (on the saved data, see this section) will have a TRUE for the clicked object from current frame to the end. *SHORTCUT: R key*

Usage: first *activate the button* (left-click or shortcut) and then *RIGHT-click* on the requested object. Repeat to undo from that frame onwards.

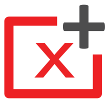

Add a delete ROI: used to add a rectangular area to automatically delete all objects contained in or touched by it.

Usage: click on the button to add a ROI. **Resize and move** the ROI to either **delete** objects or **restore** objects when they are not contained/touched anymore. Delete the ROI with right-click on it → Remove ROI.

*NOTE: Objects are **permanently deleted** only when you save AND close the GUI.*

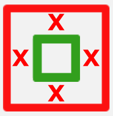

Remove segmented objects touching the border

Usage: click on the button to delete objects that are touching the border of the image

Cell cycle annotations toolbar

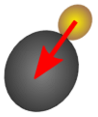

Assign bud/sister to **mother/sister**. *SHORTCUT: A key*

Usage: first *activate the button* (left-click or shortcut) and then *drag-and-drop* motion with *RIGHT-button.* Right-click on bud and release on mother, or **right-click on bud** then **right-click on mother**.

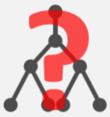

Annotate that a cell does **not** have a **fully known history.** *SHORTCUT: U key*

Usage: first *activate the button* (left-click or shortcut) and then *RIGHT-click* on the requested object.

***Tip**: two examples of cells with uknown history are cells already present at the first frame and cells appearing from outside the field of view*

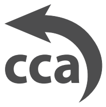

Reinitialize cell cycle annotations to default. Default is all cells in **G1** without a relative assigned to it.

Functions activated from the menus

File → Load fluorescent images...

Used to **load** as many **additional images** (e.g., fluorescence signal) as you want.

Loaded images will be used to calculate metrics such as mean, median, total amount etc. See this section for more details.

Edit → Smart handling of enabling/disabling tracking

The GUI has built-in **automatic tracking** for **time-lapse data** with the following behaviour:

- If tracking is active (Disable tracking checkbox on the toolbar is UNCHECKED) when you visit a frame that you have **never visited before**, objects will be **automatically tracked** compared to previous frame.
- When you visit a frame **already visited** before, it will **not** be **tracked**.

You can disable this automatic behaviour by unchecking `Smart handling of enabling/disabling tracking`

When you **disable** the **smart** handling, you can **enforce tracking** on all visited frames no matter if they were previously visited or not. To enforce, use the `Disable tracking` checkbox on the toolbar.

Tip*: useful when you know you have to repeat tracking on already visited frames.*

Image → Normalise intensities → ...

You can choose to **normalise the intensities** of the displayed images (saved data will not be modified) with the following methods:

- Do not normalise. Display **raw image**.
- Convert to **floating point** format with values [0, 1] → simply convert to floating point, no normalisation involved.
- **Rescale** to [0,1] → intensities are first converted to floating point if needed and then STRETCHED to convert the entire [0,1] range.
- **Normalize by max** value: divide by the max of the intensities.

Additional functions

- `Middle-click` (scrolling wheel) on Windows, `Cmd+Click` on macOS → delete the segmented object you click on
- `H` key → automatic zoom on the segmented objects
- `Double press H` key → zoom out

- `Ctrl+P` → visualize cell cycle annotations in a table
- `Ctrl+L` key → relabel object IDs sequentially (1,2,3...etc)
- `Spacebar` key → hide/show contours or segmentation masks on left image
- `Ctrl+F` key → search and highlight specific object ID
- `Alt+Click+Drag` → pan/move image

Cell-ACDC output data

Files saved by Cell-ACDC for a **fully analysed experiment** (example with original raw microscopy file called `Example1` and first position `_s01_`)

NOTE: not all files are always present, it depends on whether you have 3D data, time-lapse data, or you aligned or cropped.

`Example1_s01_acdc_output.csv`	Main table containing **cell cycle annotations** and additional **metrics** such as mean, median etc. for all the loaded channels plus all the **region properties** (as calculated by [`skimage.measure.regionprops`]) for each segmented object.
`Example1_s01_act1.tif`	.tif file for the channel called `act1`
`Example1_s01_cdc10.tif`	.tif file for the channel called `cdc10`
`Example1_s01_last_tracked_i.txt`	**Last visited frame** in "Segmentation and Tracking mode" with the main GUI
`Example1_s01_metadata.csv`	Table containing the **metadata** such as number of frames, number of z-slices, pixel size etc.
`Example1_s01_phase_contr.tif`	.tif file for the channel called `phase_contr`
`Example1_s01_segm.npz`	**Segmentation labels.** This is a numpy array (compressed).
`Example1_s01_segmInfo.csv`	Table containing information such as which **z-slice** or **z-projection** was used for segmentation or saving metrics of each channel.
`Example1_s01_align_shift.npy`	Numpy array containing the **shifts** applied to each frame when **aligning**. Useful for reverting to non-aligned state.
`Example1_s01_dataPrepROIs_coords.csv`	Table containing the **coordinates of the ROI** that was used to either **crop**, or **segment** only in the ROI. This is created in the data prep or segmentation stage.

`Example1_s01_phase_contr_aligned.npz`	Aligned data for the channel called `phase_contr`. This is a numpy array (compressed).
`Example1_s01_phase_contr_aligned_bkgrRoiData.npz`	**Data** from the **background ROIs** generated at the data prep stage.
`Example1_s01_phase_contr_dataPrep_bkgrROIs.json`	**Coordinates** of the **background ROIs** generated at the data prep stage

Adding segmentation models to the pipeline

Adding segmentation models requires few minutes:

1. Create a new folder with the model's name (e.g., YeastMate) inside the `/cellacdc/models` folder

   NOTE: if you don't know where Cell-ACDC was installed, open the main Launcher and click on the Help ➔ About Cell-ACDC menu on the top menu bar.

2. Create a `__init__.py` file inside the model folder. In this file you can handle automatic installation of the module required by your model. For example, if your model requires the module `tensorflow` you can install it manually, or programmatically as follows:

```
import os
import sys

try:
    import tensorflow
except ModuleNotFoundError:
    subprocess.check_call(
        [sys.executable, '-m', 'pip', 'install', 'tensorflow']
    )
```

   Add any line of code needed to initialize correct import of the model.

3. Create a new file called `acdcSegment.py` in the model folder with the following template code:

```
import module1
import module2

class Model:
    def __init__(self, **init_kwargs):
        script_path = os.path.dirname(os.path.realpath(__file__))
        weights_path = os.path.join(script_path, 'model', 'weights')

        self.model = MyModel(
            weights_path, **init_kwargs
        )

    def segment(self, image, **segment_kwargs):
        lab = self.model.eval(image, **segment_kwargs)
        return lab
```

   Have a look at the already implemented models (YeaZ, Cellpose, and YeastMate) as an example.

That's it. Next time you launch the segmentation module (or from the GUI menu `Segment`) you will be able to select your new model.

The model parameters will be automatically inferred from the class you created in the `acdcSegment.py` file, and a widget with those parameters will pop-up. In this widget you can set the model parameters (or press Ok without changing anything if you want to go with default parameters).

Adding trackers to the pipeline

Adding trackers requires few minutes:

1. Create a new folder with the trackers's name (e.g., YeaZ) inside the `/cellacdc/trackers` folder

   NOTE: if you don't know where Cell-ACDC was installed, open the [main Launcher]() and click on the Help➔ About Cell-ACDC menu on the top menu bar.

2. Create a `__init__.py` file inside the model folder. In this file you can handle automatic installation of the module required by your tracker. For example, if your tracker requires the module `btrack` you can install it manually, or programmatically as follows:

```
import os
import sys

try:
    import btrack
except ModuleNotFoundError:
    subprocess.check_call(
        [sys.executable, '-m', 'pip', 'install', 'btrack']
    )
```

   Add any line of code needed to initialize correct import of the model.

3. Create a new file called `trackerName_tracker.py` (e.g., `YeaZ_tracker.py`) in the tracker folder with the following template code:

```
import module1
import module2

class tracker:
    def __init__(self):
        '''here put the code to initialize tracker'''

    def track(self, segm_video, signals=None, export_to=None):
        '''here put the code to that from a segmented video
           Returns the tracked video
        '''
        return tracked_video
```

Have a look at the already implemented trackers (YeaZ, CellADCD, and BayesianTracker) as an example.

That's it. Next time you launch the segmentation module you will be able to select your new tracker.

Downstream analysis

Overview

We provide a notebook for downstream analysis called *cell_cycle_analysis.ipynb* which is meant as a starting point to generate all kinds of visualizations of the data generated by Cell-ACDC.

To **start** the notebook, open a terminal and navigate to the Cell-ACDC folder. Then type `jupyter-lab`.

This should open the project folder in the jupyter notebook interface JupyterLab. In the interface, navigate into the folder `notebooks/` and open *cell_cycle_analysis.ipynb.*

If we now switch to the “Table of contents” panel in the left, we get an overview over the notebook’s contents:

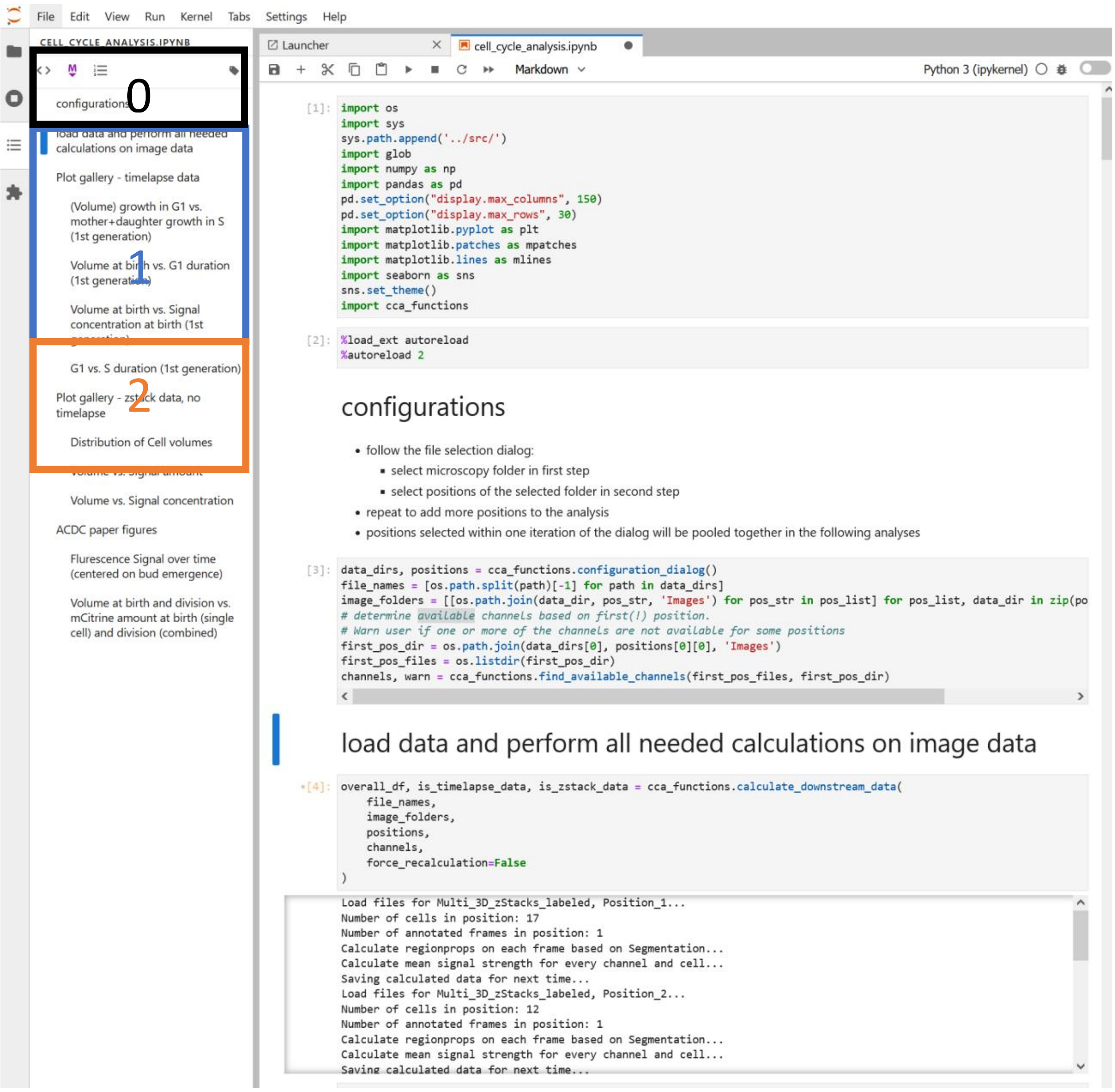

Depending on your input data, you can generate plots based on timelapse data (1) or zStack data (2) which does not contain data over time. In any case, you should run the **configuration and data load step (0)** before.

Configuring the analysis

Running the configuration cell opens a widget (**possibly in the background**) where we can select the data folder containing the "*Position_n*" folders (see First steps):

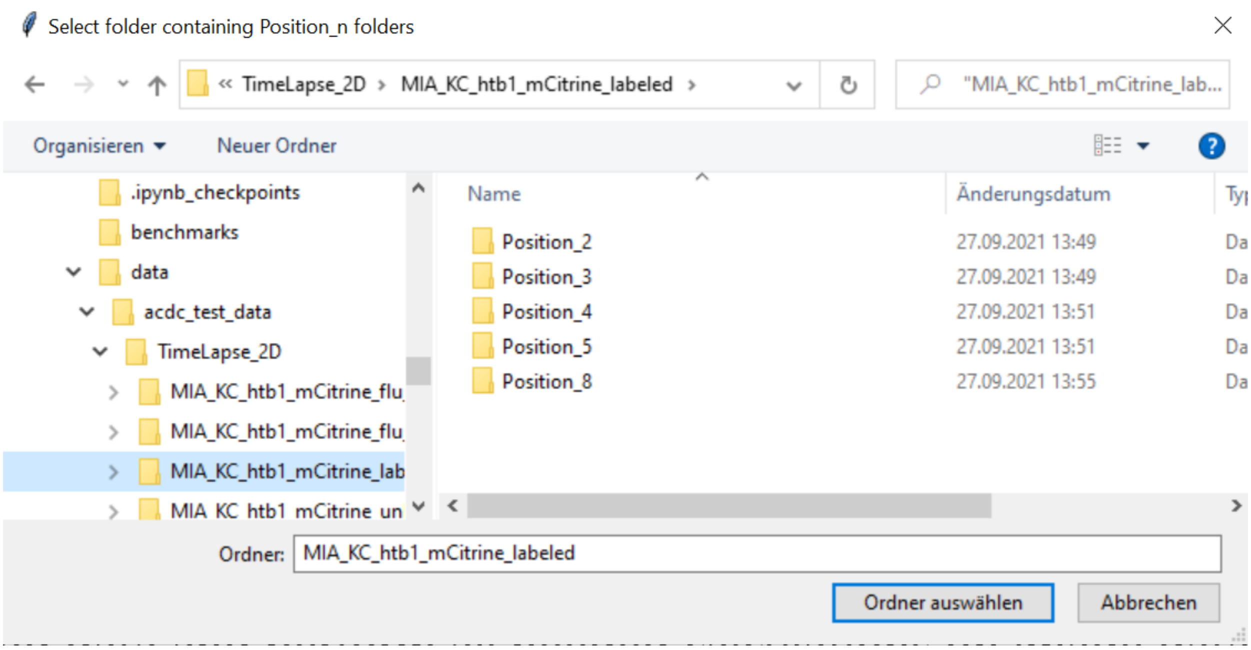

Selecting the folder leads to a window where we can select positions:

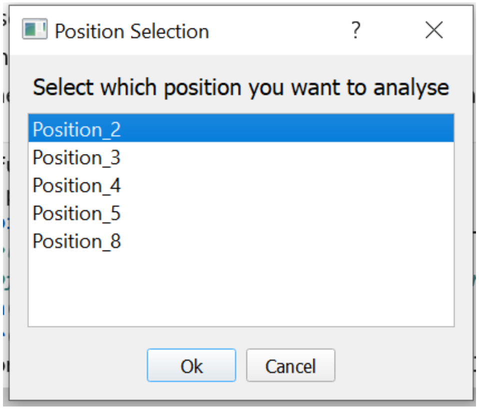

Multiple positions can be selected with `Ctrl/Shift`. All positions can be selected with `Ctrl+A`.

Next, we can select if we want to add another file and the corresponding positions to the analysis:

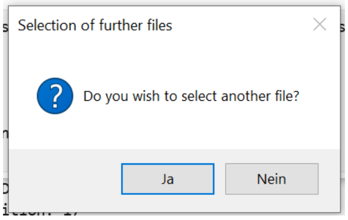

Additional files added when choosing “Yes” here, will be added to a second/third/… pool which can be helpful for comparing multiple experiments in the notebook’s plots. Choosing “No” here concludes the file selection and configuration of the notebook.

Pre-processing

Pre-processing is performed by executing the next two cells:

```
[*]: overall_df, is_timelapse_data, is_zstack_data = cca_functions.calculate_downstream_data(
         file_names,
         image_folders,
         positions,
         channels,
         force_recalculation=True
     )
```

```
Number of annotated frames in position: 241
Calculate regionprops on each frame based on Segmentation...
100%|██████████████████████████████| 241/241 [00:19<00:00, 12.38it/s]
Calculate mean signal strength for every channel and cell...
100%|██████████████████████████████| 28/28 [00:02<00:00, 12.03it/s]
Saving calculated data for next time...
Load files for MIA_KC_htb1_mCitrine_labeled, Position_4...
Number of cells in position: 54
Number of annotated frames in position: 241
Calculate regionprops on each frame based on Segmentation...
 61%|██████████████████            | 146/241 [00:11<00:09,  9.93it/s]
```

```
[*]: # if cell cycle annotations were performed in ACDC, extend the dataframe by a join on each cells relative cell
     if 'cell_cycle_stage' in overall_df.columns:
         overall_df_with_rel = cca_functions.calculate_relatives_data(overall_df, channels)
     # If working with timelapse data build dataframe grouped by phases
     group_cols = [
         'Cell_ID', 'generation_num', 'cell_cycle_stage', 'relationship', 'position', 'file',
         'max_frame_pos', 'selection_subset', 'max_t'
     ]
     # calculate data grouped by phase only in the case, that timelapse data is available
     if is_timelapse_data:
         phase_grouped = cca_functions.calculate_per_phase_quantities(overall_df_with_rel, group_cols, channels)
         # append phase-grouped data to overall_df_with_rel
         overall_df_with_rel = overall_df_with_rel.merge(
             phase_grouped,
             how='left',
             on=group_cols
         )
         phase_grouped.head()
```

Data is calculated if `force_recalculation` is set to `True` or if there is no pre-calculated data to load. After calculation, the data gets saved and automatically loaded the next time the notebook is started. By default, a check is performed and data is loaded from disk if it is already available (`force_recalculation=False`).

Plot gallery – Time-lapse data

Once pre-processing is finished, plots in the gallery can be produced by executing the corresponding cells:

Volume at birth vs. Signal concentration at birth (1st generation)

[12]:
```
# set channel name here:
ch_name = 'mCitrine'
# obtain table where one cell cycle is represented by one row:
# first set of columns (like phase_length, growth...) for G1, second set of cols for S
plot_data4 = phase_grouped[phase_grouped.cell_cycle_stage=="G1"]
plot_data4 = plot_data4[plot_data4.complete_phase==1]
plot_data4 = plot_data4[plot_data4.generation_num==1]

sns.set_theme(style="darkgrid", font_scale=2)
# Initialize the figure
g = sns.lmplot(x="phase_volume_at_beginning", y=f"phase_{ch_name}_concentration_at_beginning", data=plot_data4,
    hue="selection_subset", height=10, )
g._legend.set_title('Position Pool')
g.set(yscale="log")
ax = plt.gca()
ax.set_ylabel("mCitrine signal amount per volume in cell [a.u.]", fontsize=20)
ax.set_xlabel("Volume at birth (first cytokinesis) [fL]", fontsize=20)
ax.set_title("Volume at birth vs mCitrine signal amount per volume (1st generation)", fontsize=30)
plt.show()
```

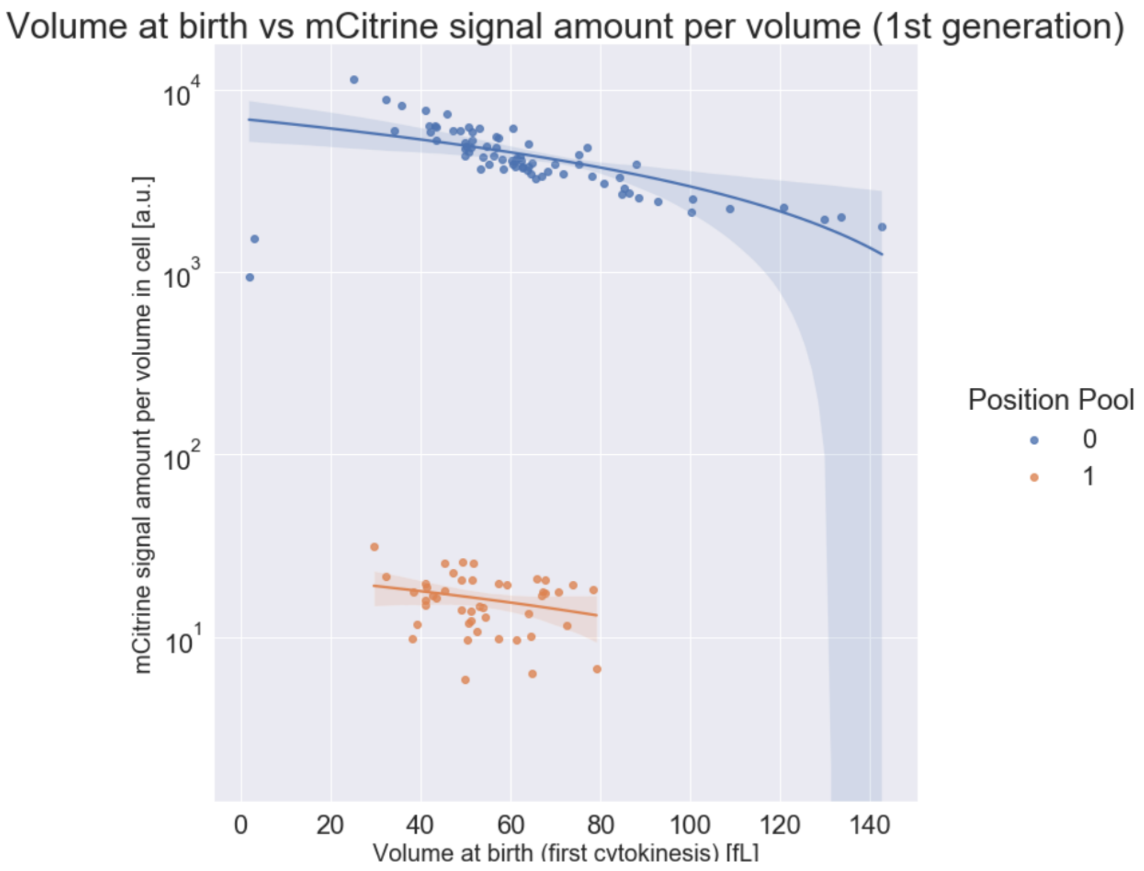

This example shows the *Htb1*-mCitrine amount per volume on the y axis and the volume at birth on the x-axis. Clearly the tagged strain (position pool 0) shows higher signal amounts as the auto-fluorescent control showing almost no signal.

Plot gallery – 3D z-stacks data

Also 3D stacks can be loaded into the downstream analysis notebook and visualized in various ways:

```
[9]: sns.set_theme(style="ticks", font_scale=2)

     # Initialize the figure
     plt.figure(figsize=(10,10))
     sns.histplot(
         x='cell_vol_fl',
         data=overall_df,
         hue='relationship',
         bins=20,
         legend=False
     )
     ax = plt.gca()
     labels = [
         'Mother cells',
         'Buds'
     ]
     handles = [
         mpatches.Patch(color=sns.color_palette('pastel')[0]),
         mpatches.Patch(color=sns.color_palette('pastel')[1])
     ]
     ax.legend(
         handles=handles,
         labels=labels,
         loc='upper right',
         #bbox_to_anchor = (1,0.2),
         framealpha=0.5
     )

     # Tweak the visual presentation
     ax = plt.gca()
     ax.set_xlabel("Cell volume [fL]", fontsize=20)
     ax.set_title(f"Volume distribution, n: {overall_df.shape[0]}", fontsize=30)
     #sns.despine(trim=True, left=True)
     plt.show()
```

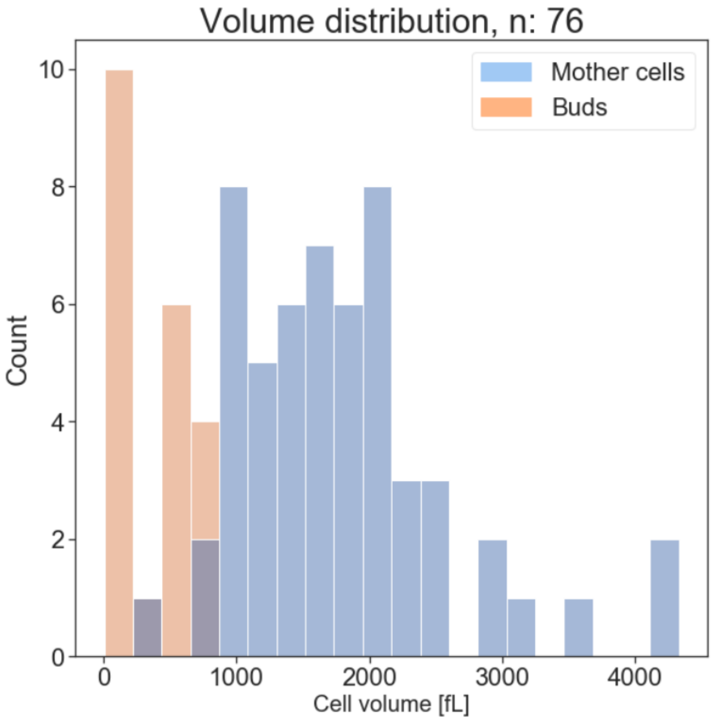

Unsurprisingly, the buds in the test position show way smaller volumes than mother cells. Outliers like the mother cell showing a very small volume in this case, can be identified and checked in the GUI.
